# Nanomaterial-Enabled Sensors and Therapeutic Platforms for Reactive Organophosphates

**DOI:** 10.3390/nano11010224

**Published:** 2021-01-16

**Authors:** Seok Ki Choi

**Affiliations:** 1 Michigan Nanotechnology Institute for Medicine and Biological Sciences, University of Michigan Medical School, Ann Arbor, MI 48109, USA; skchoi@umich.edu; 2 Department of Internal Medicine, University of Michigan Medical School, Ann Arbor, MI 48109, USA

**Keywords:** reactive organophosphate, oxime, antidote delivery, nanosensor, nanoscavenger

## Abstract

Unintended exposure to harmful reactive organophosphates (OP), which comprise a group of nerve agents and agricultural pesticides, continues to pose a serious threat to human health and ecosystems due to their toxicity and prolonged stability. This underscores an unmet need for developing technologies that will allow sensitive OP detection, rapid decontamination and effective treatment of OP intoxication. Here, this article aims to review the status and prospect of emerging nanotechnologies and multifunctional nanomaterials that have shown considerable potential in advancing detection methods and treatment modalities. It begins with a brief introduction to OP types and their biochemical basis of toxicity followed by nanomaterial applications in two topical areas of primary interest. One topic relates to nanomaterial-based sensors which are applicable for OP detection and quantitative analysis by electrochemical, fluorescent, luminescent and spectrophotometric methods. The other topic is directed on nanotherapeutic platforms developed as OP remedies, which comprise nanocarriers for antidote drug delivery and nanoscavengers for OP inactivation and decontamination. In summary, this article addresses OP-responsive nanomaterials, their design concepts and growing impact on advancing our capability in the development of OP sensors, decontaminants and therapies.

## 1. Introduction

**Figure 1:**
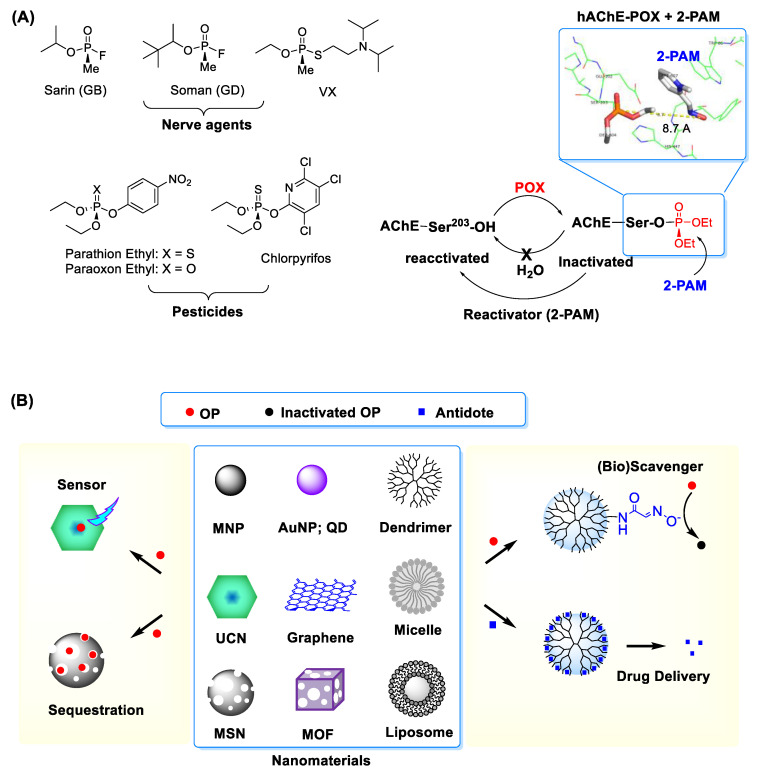
(**A**) (left) Types of representative organophosphates (OP) including nerve agents and pesticides and (right) covalent inhibition of acetylcholine esterase (AChE) by OP such as paraoxon ethyl (POX). Inset: An X-ray crystal structure for POX-inactivated human AChE in complex with 2-PAM, an enzyme reactivator which works by nucleophilic dephosphorylation (protein data bank (PDB) code 5HFA [10]). Adapted with permission from [16], Copyright 2019, The Royal Society of Chemistry. (**B**) Selected nanomaterials and their applications in OP detection and treatment. Abbreviations: MNP = magnetic nanoparticle, AuNP = gold (Au) NP, QD = quantum dot, MSN = mesoporous silica nanoparticle, MOF = metal-organic framework, UCN = upconversion nanocrystal.

The term reactive organophosphates (OPs) refers collectively to a group of phosphorous-based toxic chemicals that cause life-threatening toxic symptoms in humans. These comprise OP nerve agents such as sarin, soman and VX, as well as OP-based pesticides like chlorpyrifos, paraoxon and malaoxon, among others (Figure 1) [1,2,3]. Despite their much lower toxicity, the OP pesticides are still formidable due to their wide distribution ranging from insect controls in the agricultural and horticultural sectors to pest treatments for domestic pets, farm animals and houses [4]. OP toxicity is commonly attributed to a phosphorous (thio)ester core that serves as its reactive functionality. It is highly susceptible to engaging in a covalent conjugation with a nucleophilic residue present in proteins and cellular enzymes in plasmas [1,5,6,7,8]. This OP reaction results in a covalent protein modification and thus loss of their original activity.

Human toxicity induced by OPs is attributed to the inactivation of acetylcholinesterase (AChE) as their primary target [1,5,6,7,8]. Human AChE is expressed widely from red blood cells, muscle tissues to nervous systems [9]. Its inactivation by OPs involves a covalent adduct formed within its catalytic pocket through O-phosphorylation at serine 203, one of catalytic residues (Figure 1) [10]. Such enzyme inactivation contributes to acetylcholine accumulation, which in turn leads to its excessive stimulatory activity at cholinergic receptors in nervous systems [10,11]. Their pathophysiological effects are linked to induction of rapid muscle paralysis and occurrence of various neurological symptoms, both constituting the toxicological basis of OPs [12,13,14]. Their toxic syndromes include respiratory problems, paralysis, convulsions, salivation, gastric cramps and emesis whereas their manifestations are determined by various factors such as OP types, receptors stimulated, organs affected, action sites and time of occurrence (acute, delayed) [15]. All these make effective treatment of OP intoxication and optimal timing of patient discharge highly challenging.

OP exposures remain a source of serious safety concerns given the history of accidental or terrorist incidents and increasingly indiscriminate use of OP pesticides that is responsible for environmental pollution and crop contamination [13]. In particular, persistent exposures to OP pesticides result in delayed or chronic toxicity in affected organisms [2,3]. This longer-term effect is closely related with their environmental disposition and fate which occurs via soil adsorption, distribution in groundwater and volatilization into air [2,5,6]. The key process that determines their fate involves OP degradation to harmless fragments through chemical hydrolysis, microbial degradation and photolytic breakdown under sunlight irradiation. Most OP pesticides show stability in water at neutral pH [2,5], but their hydrolytic breakdown occurs faster under non-neutral conditions or elevated temperatures [2,6,7,8]. The extent of OP biodegradation is largely dependent on microbial species and their mechanisms of biotransformation which involve *O*-dealkylation by glutathione *S*-transferase, oxidation of thioether to sulfoxide, desulfuration, hydrolysis, and reduction [9,10]. Light exposure is another factor that plays a crucial role in the degradation pathway of OP pesticides [11,12]. The extent of photolysis varies with OP types, light wavelength and intensity associated with solar radiation [12,13]. In summary of environmental exposure, OP intoxication can occur through various routes including oral consumption, skin contact [17] or inhalation of OP sprayed in the air [18]. These contribute to either acute, delayed or chronic toxicity upon exposure [19]. However, despite such concerns, current capability to address OP issues remains suboptimal due to paucity of advanced technologies that enable for sensitive OP detection or effective treatment.

Nanomaterials have made a growing impact on developing therapeutic agents [20,21,22,23,24,25,26,27] and sensors [28] in numerous areas. These are classified as a group of objects or structures in a nanometer size range which display functional properties distinct from bulk materials [29,30]. Such properties are characterized largely by their composition, size and shapes, which include nanospheres [31,32], nanotubes [33], planar nanosheets [34], nanodisks [34,35], nanocages [36] and nanorods [32] (Figure 1). They allow structural modifications and offer functional capabilities through magnetic control, light absorption, fluorescence, luminescence, cavity loading or pore gating. These properties make nanomaterials highly applicable for sensors [36], drug delivery platforms [20] or nanoscale reactors [37,38]. Therefore, nanomaterials applications offer a high potential to address existing OP-related serious problems.

This article aims to review the status and advances of such OP-responsive nanomaterials and enabling nanotechnologies. It is directed on two primary areas of application: (i) sensors, (ii) therapeutic development for OP exposure and intoxication. The first relates to nanosensor types and capabilities applicable for OP detection through instrumental analysis. The second describes nanomaterial applications in the development of antidote delivery platforms and nanomaterial-based OP scavenges which are designed through bioscavenger encapsulation or chemical functionalization. In summary, this article hopes to serve a concise yet updated review on OP-responsive nanomaterials, their design concepts and impactful applications.

## 2. Nanosensors for Reactive Organophosphate Detection

### 2.1. Electrochemistry

#### 2.1.1. AChE-Immobilized Electrode

Electrochemical detection constitutes one of fundamental approaches in biosensor design for OP analysis [39]. This often relies on fabricating an OP-responsive electrode through its surface functionalization such as by immobilization with AChE [39,40,41,42]. This enzyme functionalization is therefore responsible for generating an OP-specific signal in amperometry or voltammetry when its immobilized enzyme loses its catalytic activity upon inactivation by OP [39,40,41]. This detection method is validated for its ability to detect individual OP pesticides or their mixture.

#### 2.1.2. AChE-Immobilized Nanosensor

In an electrochemical nanosensor design, AChE is immobilized on the nanoparticle (NP) surface in lieu of the bulk electrode surface. This approach has been applied to magnetic nanoparticles (MNPs) such as iron oxide (Fe_3_O_4_) nanoparticle (IONP) [43] and nano Fe-Ni [44], each offering an important benefit of magnetic control. Thus, using AChE-immobilized MNPs allows temporal and spatial control of MNP localization in an working electrode or screen-printed electrode under an applied magnetic field [43] as reported by Rodrigues et al. In this study, they report unique benefits such as ability for nanosensor assembly on demand and convenience in electrode renewal (cleaning). These are otherwise not available simply by permanent AChE immobilization on the electrode surface. AChE immobilization in MNP-based nanosensors can be achieved by protein crosslinking through glutaraldehyde [43], Ni-histidine tag [45,46] or light responsive polymer [45]. Their sensitivity for OP detection is validated with pesticides such as chlorpyrifos and malathion with limit of detection (LOD) as low as sub nM (Table 1).

Non-magnetic NPs are also employed in developing electrochemical nanosensors. These include gold nanoparticle (AuNP) [47], nano Ag [48] and mesoporous silica nanoparticle (MSN) [49], each functionalized by AChE immobilization or non-covalent encapsulation for OP specificity. These nanosensors offer sufficient sensitivity to detect a wide range of OP pesticides as listed in Table 1.

#### 2.1.3. Antibody-Immobilized Nanosensor

Another approach for OP detection involves using an antibody raised against a specific OP species. This is described in a study reported by Mehta et al. [50] in which an anti-parathion antibody was immobilized on the surface of graphene nanosheet (GNS). As a two-dimensional nanostructure, GNS displays an excellent degree of conductance for electrons, which is hence highly suited for application in electrochemical biosensing. This GNS-based immunosensor showed high detection sensitivity for parathion or parathion-like pesticides with LOD as low as fM [50]. However, despite such sensitivity, using an immunosensor has certain drawbacks because its employed antibody is able to recognize only a specific subset of OPs and not applicable to a broader spectrum of OPs [50].

#### 2.1.4. OP-Responsive Nanosensor

**Figure 2:**
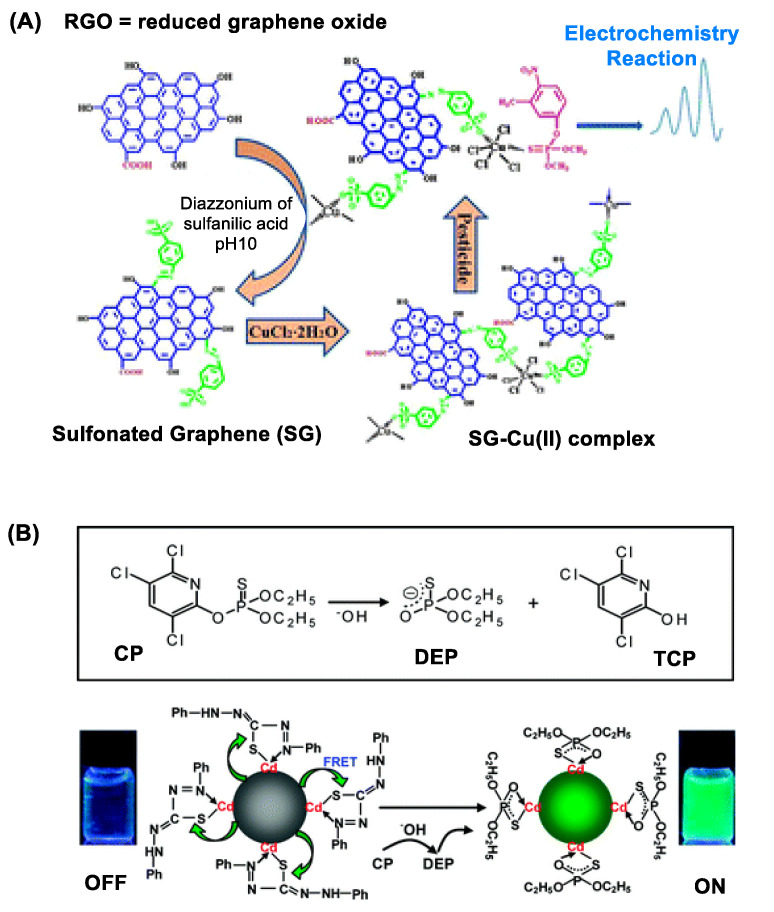
(**A**) A copper (II)-functionalized graphene nanocomposite applied for sensing sulfur-containing organophosphate (OP) pesticides. Reproduced with permission from [51], Copyright 2013, The Royal Society of Chemistry. (**B**) Dithizone-coordinated CdTe quantum dot (QD) applied for chlorpyrifos detection. Its concept of detection involves restoration of its fluorescence by dithizone replacement with diethylphosphorothioate, a hydrolytic byproduct of chlorpyrifos. Reproduced with permission from [56], Copyright 2010, American Chemical Society.

As introduced briefly above, GNS display unique features in its structure and property beneficial for electrochemical OP detection. These include high surface area-to-volume ratio, ultralow thickness and high electronic conductance [65]. Their combination confers GNS with sensitive ability to respond to OP adsorption or reaction that occurs on its surface. This is illustrated with a copper-graphene nanocomposite in Figure 2A that shows ability to detect sulfur-containing OP pesticides [51]. Such GNS-based OP detection is further validated using copper-coated reduced graphene oxide (rGO) [51], AuNP-coated rGO [52] and AuNP-coated GNS [53]. Besides, GNS nanosensors are designed by surface modification with an OP-specific probe molecule that engages in selective OP recognition and/or its reaction. Huixiang et al. [54] validated this concept using GO@AuNP functionalized with 4-aminoacetophenone oxime. Thus, an electrode fabricated with this graphene nanocomposite has led to OP detection with LOD at low nM (Table 1).

In summary, electrochemical nanosensors have shown promising capabilities for OP detection. These are designed with nanomaterials such as IONP [43,45,46], nano Fe-Ni [44], AuNP [47], nano Ag [48], MSN [49] or graphene-based NP [51,52,53,54], each functionalized with AChE [43,44,47,48,49], OP antibody [50] or OP-reactive moiety [54]. These nanosensors offer characteristic advantages including high loading capacity in electrodes, high sensitivity, and fast onset of action due to a narrow spacing between interacting electrodes. Their capabilities are attributable to a combination of their nanometer size, shape and other design features which are not available by conventional bulk electrodes [3,43].

### 2.2. Absorbance, Fluorescence and Luminescence Spectroscopy

In general, most OPs do not contain chromophores that are applicable for spectroscopic detection by UV–Vis absorbance or fluorescence. There are only few OPs which contain aromatic moieties for UV absorbance such as parathion, paraoxon (POX) and fenitrothion [66], each containing a 4-nitrophenyl group. However, their direct detection by spectrometry is not efficient because their molar absorptivity is practically too low for sensitive analysis. Instead, these are better detectable indirectly through a mechanism of fluorescence quenching in which each chromophore serves as a fluorescence quencher to a sensor molecule added separately such as coumarin [67]. This fluorescence quenching assay is validated with parathion, POX and fenitrothion, and it displays relatively low sensitivity in the range of 10^−7^–10^−4^ M [66].

#### 2.2.1. Quantum Dot (QD) Nanosensors

QDs are notable for their bright fluorescence in the visible and near infrared (NIR) range [56,68]. Their fluorescence is applicable for OP detection as illustrated with QD sensors made of CdTe [55,56], CdS [60] and Mn-doped ZnS [57]. Their detection principle varies with specific design features introduced in each sensor, but it involves measuring a change in QD fluorescence intensity that occurs in response to OP adsorption or a chemical reaction on the QD surface [55]. The change occurs via either fluorescence resonance energy transfer (FRET) [56,60] or photoelectron transfer (PET) [57] between the donor (QD) and the OP-responsive acceptor attached on the surface. Zhang et al. reported dithizone-coordinated CdTe QD designed for FRET quenching-based chlorpyrifos detection via dithizone hydrolysis as shown in Figure 2B [56].

#### 2.2.2. Upconversion Nanocrystal (UCN) Nanosensors

**Figure 3:**
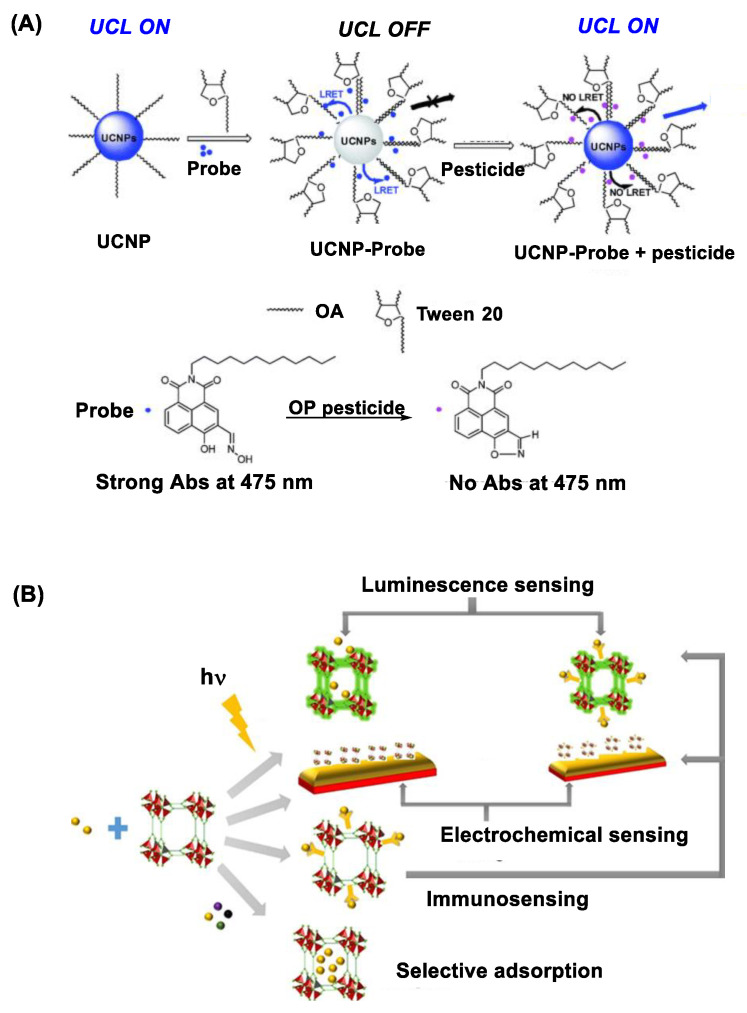
(**A**) Upconversion nanocrystal (UCN) immobilized with an oxime probe on the surface applied for OP detection through the mechanism of luminescence resonance energy transfer (LRET) between UCN and the oxime probe involved in organophosphate detection. Reproduced with permission from [61], Copyright 2016, The Royal Society of Chemistry. (**B**) A schematic diagram for metal-organic framework (MOF)-based approaches developed for pesticide sensing. Reproduced with permission from [72], Copyright 2018, American Chemical Society.

UCNs belong in an emerging class of photoactive nanomaterials that include NaYF_4_ doped with lanthanide ions (Yb, Er, Tm) in their lattice structure [69,70]. Unlike QDs, UCNs are excited by irradiation at longer NIR wavelengths (980 or 808 nm) with ability to emit upconversion luminescence at shorter visible wavelengths such as 475 nm [71]. Their luminescence intensity is sensitive to surface functionalization, and it can be quenched via its luminescence resonance energy transfer (LRET) to an acceptor molecule localized at a close proximity. In a recent study, Wang et al. [61] describes such luminescence quenching using UCN functionalized with an OP-reactive oxime probe on the surface (Figure 3A). This quenched luminescence is applicable for OP detection because it is restored when the oxime probe reacts with OP which leads to LRET deactivation. This UCN-based nanosensor has shown a detection sensitivity for diethyl chlorophosphate or dimethoate at μM [61]. 

#### 2.2.3. Metal-Organic Framework Nanosensors

OP can be detected by metal-organic frameworks (MOFs) that are active in UV photoluminescence. These include MOFs made of luminescent transition metal or lanthanide ions coordinated to organic ligands (imidazole) [73,74]. Their photoluminescence is highly responsive to microenvironmental changes in their lattice structure such as binding by guest molecules. Thus it is diminished to a significant extent upon OP binding or encapsulation (Figure 3B) [72]. This MOF-based luminescence assay enables to detect a broad spectrum of OP pesticides including chlorpyrifos, parathion and azinphos-methyl as described in a study by Singha et al. [75]. MOF sensors can be tunable in their design for improved guest specificity as reported with hafnium (Hf) ion-doped MOF [58,76]. In this study, Lian et al. describes its specific response to methanephosphonate, a hydrolytic byproduct from nerve agents, with high sensitivity. Other design factors in MOF sensors include those related to addressing potential drawbacks such as suboptimal aqueous stability, relatively slow onset of response and signal interference by other chemicals [72]. 

#### 2.2.4. Plasmonic Nanomaterials

Noble metal nanomaterials that include nano gold (Au) or nano silver (Ag) display light absorbance via surface plasmon resonance (SPR) in the range of 350–500 nm (nano Ag) and 450–600 nm (nano Au) [32,77]. Their SPR absorbance is applicable for OP detection because it makes a blue shift upon chemisorption by sulfur analytes such as thiol-releasing OPs or thion (P=S)-based OPs (Table 1) [63]. Their detection sensitivity varies with metal compositions and shapes as evident with hexagon-shaped nano Ag which detects chlorpyrifos more effectively than other shapes [64].

Development of plasmonic nanosensors based on nano Au and nano Ag has certain limitations because they are not directly applicable for certain OPs that lack a sulfur moiety. Such lack of broader sensitivity is however addressed by surface functionalization with an OP-specific sensing element such as AChE [62], rhodamine B [59] or adenosine triphosphate [59]. Each of these sensors, which works in a different manner, has shown a broader sensitivity extended to oxon-based ethoprophos and dichlorvos [59,62] (Table 1).

In topic summary, several types of nanosensors are developed for OP analysis with improvement in detection time, sensitivity and specificity. Their capabilities are attributable to nanoscale structural and functional properties enabled by various types of nanomaterials that include MNPs [43,45,46], nano Au [47,59], nano Ag [48], MSN [49], graphene [50,51,52,53,54], QDs [55,56,57], luminescent UCN [61] and MOF [58]. These nanosensors are applicable for instrumental OP analysis by electrochemistry, SPR absorbance, fluorescence and luminescence.

## 3. Therapeutic Platforms for Reactive Organophosphate Treatment

### 3.1. Delivery Systems for Antidotes 

Over several decades, we have seen continued and significant efforts to develop for therapeutic agents effective in the treatment of OP intoxication [12,78]. Despite such attention, current approved regimen still relies on administering one or more of relatively old antidotes [12,79]. These include oxime agents including pralidoxime chloride (2-PAM) that serves as an enzyme reactivator, atropine as a cholinergic receptor blocker and midazolam, an anticonvulsant for epileptic seizure relief [80]. Of these, 2-PAM serves a primary antidote due to its critical role in reactivating OP-inhibited AChE (Figure 1). However, 2-PAM has shortcomings such as inability to cross the blood-brain barrier needed for its central nervous system (CNS) bioavailability [81,82] and its short half-life (≤2 h) in plasma [83,84].

#### 3.1.1. Oximes

**Figure 4:**
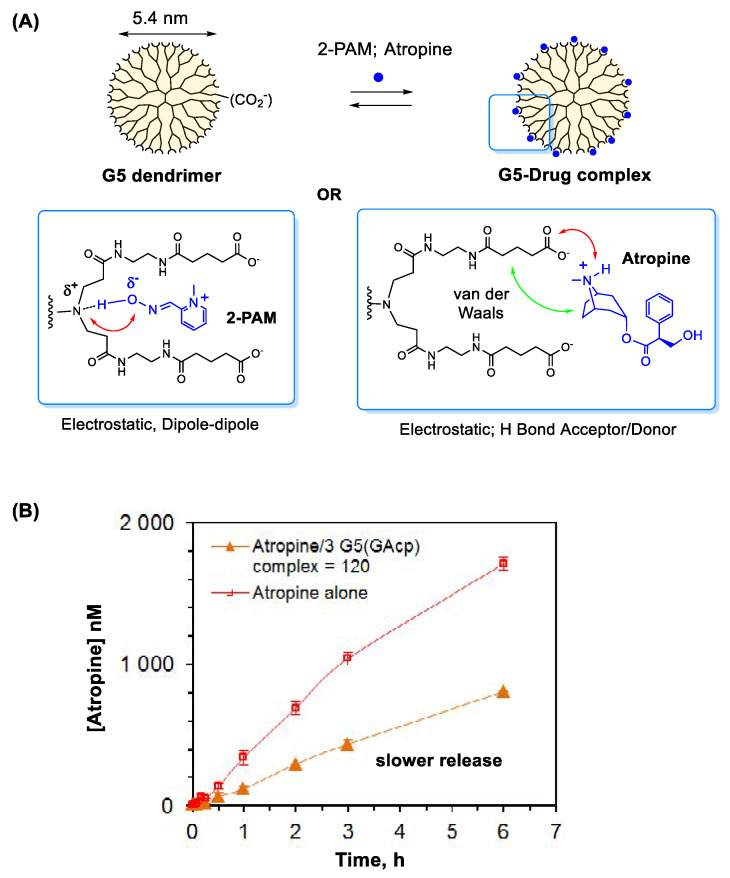
(**A**) A proposed model for 2-PAM or atropine complexation in fifth generation (G5) PAMAM dendrimer [86,89] and (**B**) a drug release kinetics for an atropine-dendrimer complex. G5(GA_cp_) refers to a G5 PAMAM dendrimer modified with cyclopentane (cp)-fused glutarate (GA). Reproduced with permission from [89], Copyright 2015, American Chemical Society.

It is well documented that nanomaterials serve as effective carriers in drug delivery [20,21]. They have shown promising potential in controlled antidote delivery as evident with systems based on MOF [85] and poly(amidoamine) (PAMAM) dendrimer [86,87], each applied in oxime delivery. These systems vary in structural features, modes of interaction applicable for drug encapsulation and loading capacity. The MOF system, which is characterized with its extensive array of internal pores, shows host-guest complexation for 2-PAM encapsulation through π-stacking and hydrogen bond interactions [85]. On the other hand, PAMAM dendrimer offers its well-defined architecture that contains hydrophobic cavities in its internal core and an array of repetitive peripheral branches, both applicable in guest complexation [88]. This unique architecture plays an essential role in 2-PAM complexation which occurs through electrostatic and hydrogen bond interactions at peripheral sites (Figure 4A) [86,87]. This type of oxime binding has been demonstrated with G5 PAMAM dendrimers, either unmodified [87] or peripherally modified with glutarate [86] as illustrated in Figure 4A. Use of such delivery systems based on MOF or dendrimer can serve an important route for extended duration of drug action because drug release from such complexes occurs more slowly than free drug [85,86,87].

**Figure 5:**
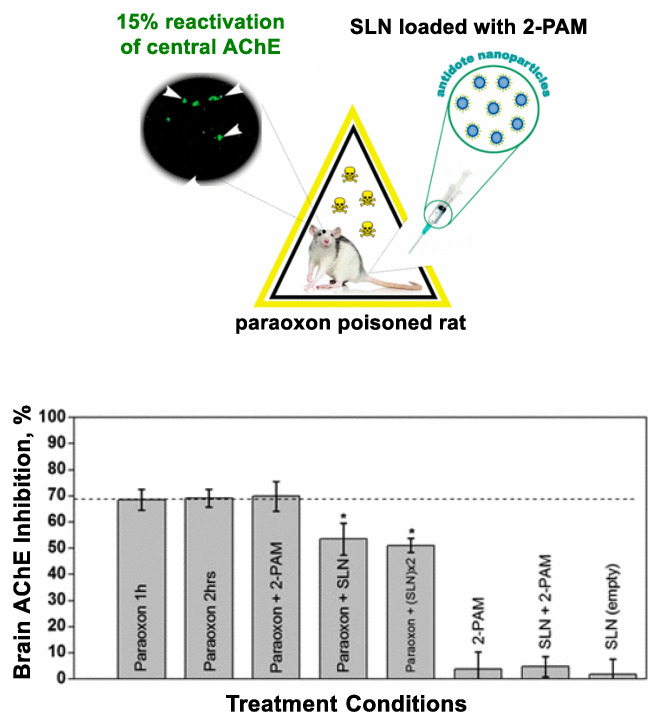
CNS drug delivery using 2-PAM-loaded solid lipid nanoparticles (SLN). This treatment shows a higher level of acetylcholinesterase (AChE) reactivation (lower inhibition) in paraoxon-poisoned rats. * statistical significance of *p* < 0.05 relative to a control (paraoxon alone). Adapted with permission from [91], Copyright 2017, American Chemical Society.

Nanocarriers are further validated in CNS drug delivery [84,90,91]. This illustrated with two systems based on either human serum albumin [90] or solid lipid nanoparticles (SLNs) [84,91], each investigated for oxime transport across the blood-brain barrier. The serum albumin nanoparticles [90] have shown oxime (obidoxime, HI-6) transport in a model CNS system to a greater extent than free oximes. SLNs have been studied otherwise for in vivo oxime delivery in rat brain as shown in Figure 5 [84,91]. In this approach, Pashirova et al. [91] designed 2-PAM-loaded SLN and demonstrated its greater efficacy in AChE reactivation (lower inhibition) in POX-poisoned rats than its free drug.

#### 3.1.2. Atropine

The delivery strategy described above for oximes is similarly applied to atropine, another essential antidote used in the treatment of OP poisoning [12]. Like 2-PAM, atropine displays a short half-life (t_1/2_ ≈ 1–2 h in human plasma) primarily due to rapid metabolism and excretion [92]. PAMAM dendrimer is validated as its delivery system. Driving forces for its complexation involve a combination of electrostatic association and hydrophobic van der Waals interaction as shown in Figure 4A [89]. Its dendrimer complex displays slower release than free atropine (Figure 4B), suggesting a potential contribution to its extended duration of action [22,93,94]. Another system studied for atropine delivery involves poly(methacrylate) polymer prepared via molecular imprinting for atropine recognition [95]. This polymer has shown its binding affinity for atropine and utility in controlled release in vitro.

### 3.2. Bioscavengers

Bioscavengers refer to a class of enzyme-based proteins that are able to recognize and inactivate OP molecules [38,96,97,98,99]. Therefore, they offer a therapeutic potential as therapeutic antidotes. They comprise a broad range of OP hydrolytic proteins that include butyrylcholinesterase (BChE) [100,101,102], carboxylesterase 1 (hCE1) [103] and paraoxonase (PON) [104,105] in human plasma and organophosphorus anhydrase [106]. Non-enzymatic proteins such as albumins can also participate in OP inactivation through stochastic reactions [107]. Like AChE, most of these enzymes belong in the class of stoichiometric (suicidal) scavengers which are inefficient due to their OP inactivation in a 1:1 molar ratio. Such low efficiency is addressed by catalytic bioscavengers [97,108,109,110] that include mutant AChE [111,112] and bacterial phosphotriesterase [109,112].

Despite their therapeutic potential, their commercial development faces challenges to overcome due to issues associated with enzyme instability, short plasma half-life and large doses required. Some of these undesired properties could be improved by conjugation strategies used in nanotechnology. These include enzyme PEGylation [38,108,113] or conjugation with an Fc protein [114], each known to prolong the duration of circulation in plasma. Another approach involves nanomaterial integration as illustrated with polymer integration (coating) with acetylcholinesterase-embedded cell membranes. This integrated system has retained AChE activity against dichlorvos in vitro and shown efficacy in protecting mice from dichlorvos [38]. Similarly, organophosphorus anhydrase (OPAA) encapsulated in a poly(carboxybetaine) polymer hydrogel has shown potent efficacy in protecting guinea pigs from repeated exposures to sarin [106].

### 3.3. Chemical Scavengers

**Figure 6:**
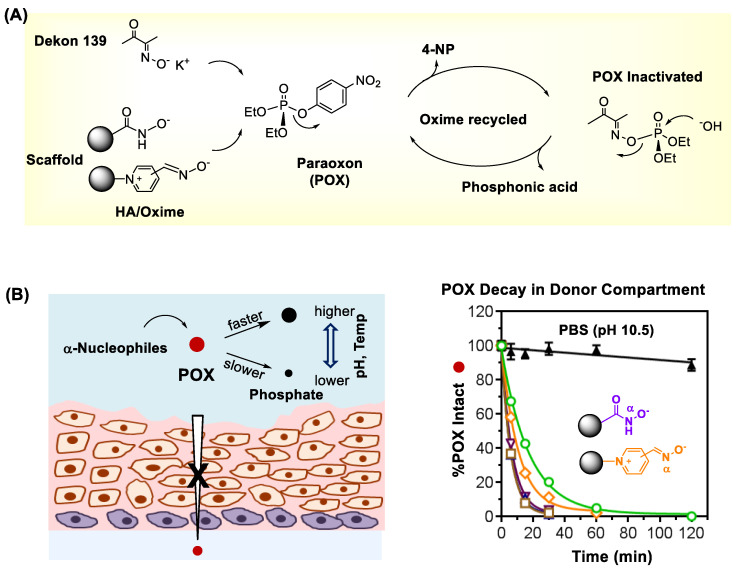
(**A**) The mechanism of hydrolytic organophosphate (OP) inactivation by oxime or hydroxamate (HA)-based scavengers as illustrated with paraoxon ethyl (POX). (**B**) Topical decontamination of porcine skin exposed to POX ex vivo by treatment with chemical scavengers Dekon 139 and oxime/HA (α-nucleophile) scaffolds (left) and decay curves of POX in the donor chamber (right). Reproduced with permission from [16], Copyright 2019, The Royal Society of Chemistry.

In addition to antidote administration, performing a topical decontamination plays an essential role in effective OP treatment. It is because without such OP removal from the skin surface, the exposed surface serves as a depot for extended OP absorption which causes sustained toxicity [115]. Several types of decontaminants [96,116,117,118,119,120,121] have been developed for skin decontamination that include most notably reactive skin decontamination lotion (RSDL) [122,123]. Its active ingredient consists of Dekon 139, a potassium salt of diacetyl monooxime (DAM). It displays a strong chemical reactivity in OP inactivation through catalytic hydrolysis as depicted in Figure 6A [124]. However, Dekon 139 can induce adverse systemic effects such as skeletal muscle paralysis due to its rapid skin and wound absorption [125].

Recently, several types of molecular scaffolds are designed and tested as effective chemical decontaminants for OP pesticides and nerve agents [119,121,126,127,128]. These include α-nucleophile scaffolds prepared by conjugation with oxime [16,124], pyridinium aldoxime (PAM) [120,129,130] or hydroxamic acid (HA) [16,129,131]. Some of these have shown greater efficiency in topical decontamination of porcine skin exposed to POX ex vivo than Dekon 139 [16,124]. This is evident with faster decay curves of POX in the donor chamber by treatment with oxime/HA scaffolds as shown in Figure 6B [16].

Other scaffolds such as those prepared by PAM conjugation to a glucose [2] or β-cyclodextrin [132,133] unit have shown potent reactivity in OP (POX, cyclosarin) inactivation in vitro [132,133] or preventing POX-induced hypothermia in vivo [2]. Non-α-nucleophile scaffolds are similarly validated as OP-reactive constructs that include alkali metal alkoxides [126], lanthanide (La^3+^) ions [121], nitrogen-containing bases [127] and imidazolium-based ionic liquids [119].

### 3.4. Nanoscavengers

#### 3.4.1. Lipid Nanoparticles

**Figure 7:**
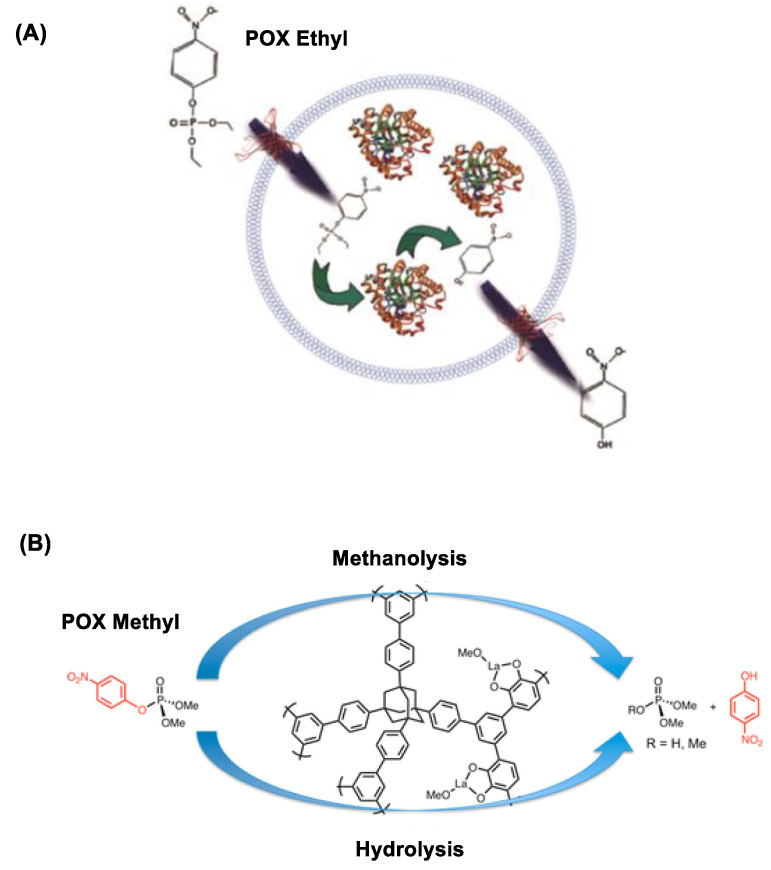
(**A**) Inactivation of paraoxon ethyl (POX) by phosphotriesterase (PTE)-encapsulated vesicles. Reproduced with permission from [139], Copyright 2018, American Chemical Society. (**B**) POX methyl inactivation by lanthanide-catechol functionalized porous organic polymer. Reproduced with permission from [140], Copyright 2013, American Chemical Society.

Micelles and liposomes have been frequently employed in the design of lipid-based nanoscavengers for OP hydrolysis [120,134,135,136]. Their typical design involves presentation of OP-reactive oxime or HA moieties on the external surface [137,138]. This is illustrated with PAM-functionalized micelles demonstrated for their catalytic role in the hydrolysis of POX, parathion and fenitrothion [135,136]. Similarly, oxime-incorporated liposomes are able to accelerate the hydrolysis of OP such as 4-nitrophenyl diphenyl phosphate by several orders of magnitude faster than free oximes [136]. Furthermore, unlike micelles, the liposomal interior serves a water-filled cargo space for (bio)scavenger loading. Thus, liposomes encapsulated with bioscavenger phosphotriesterase are demonstrated for effective POX decontamination on model surfaces as shown in Figure 7A [139].

#### 3.4.2. Metal Ion Chelated Polymer

Polymer-based scavengers are designed by polymer modifications with OP-reactive metal chelates [140,141]. These include a copper (II)–vinylbipyridyl complex chelated to trimethylolpropane trimethacrylate demonstrated for its ability to accelerate the hydrolysis of 4-nitrophenyl phosphate and parathion-methyl compared to the free metal complex [141]. Lanthanide (La) chelates likewise play an effective role in catalytic OP hydrolysis [141]. This is shown with porous organic polymer functionalized with La-catecholate residues demonstrated for faster degradation of paraoxon methyl as illustrated in Figure 7B [140].

#### 3.4.3. Mesoporous Silica Nanoparticle

MSNs consist of low nanometer-sized pores in their internal architecture [142]. Their porous spaces are highly useful for application in the physical adsorption and sequestration of smaller guest molecules [143]. Moreover, silanol groups that account for a major fraction of surface functionality can directly engage in degradation reactions of adsorbed OP molecules [144]. This is shown by MSN with a pore diameter of 2.4 nm. It is demonstrated for effective OP adsorption and detoxification against OP molecules as large as dichlorvos and dimethyl methylphosphonate [144]. 

#### 3.4.4. Metal-Organic Framework

**Figure 8:**
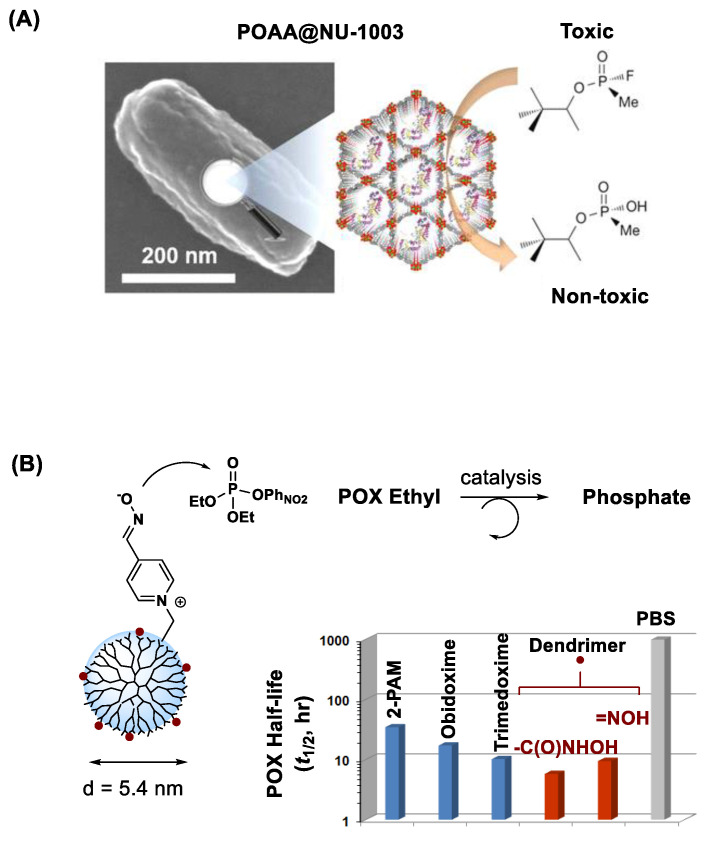
(**A**) Inactivation of soman (GD) by mesoporous zirconium-based metal–organic framework (MOF; NU-1003) immobilized with organophosphorus acid anhydrolase (OPAA). Reproduced with permission from [117], Copyright 2016, American Chemical Society. (**B**) POX inactivation by pyridinium aldoxime (PAM)-based dendrimer nanoscavengers. Reproduced with permission from [130], Copyright 2014, The Royal Society of Chemistry.

The internal architecture of MOFs offers an extensive array of nanopores [145] which are variable in diameter [145]. These pores allow to adsorb and sequester small molecule contaminants such as OP pesticides [73]. MOFs can be tunable in pore size as seen in zirconium-based MOF that offers a pore aperture large enough for immobilizing bioscavenger molecules such as OPAA as shown in Figure 8A [117]. In this study, Li et al. describes that this enzyme-immobilized MOF is highly reactive in OP hydrolysis, leading to much faster degradation of a nerve agent soman compared to the enzyme alone or the enzyme immobilized in MOF with smaller pores. Moreover, MOFs are tunable in the design of internal functionality as seen in amine-presented UiO-66-NH_2_ [146,147]. Such amine-functionalized MOF has shown reactivity in degrading POX and nerve agents [148].

#### 3.4.5. Metal Oxide Nanoparticle

Metal oxide-based nanomaterials contain a large array of chemically reactive sites on their exposed surface which are applicable for OP degradation. Of note is CeO_2_ NP reported for its successful application in preventing POX decontamination in a porcine skin model [149]. Its efficacy is attributable to its oxide functionality as the sites which engage in POX degradation. In addition, CeO_2_ NP has shown faster POX degradation under UV–Vis irradiation than an ambient light condition. Occurrence of such photocatalytic reactivity is broadly reported in other types of metal oxide NPs including TiO_2_ and Ag-ZnO [150,151]. Their photocatalytic reactivity is linked to more effective degradation of parathion and chlorpyrifos that occurs under a (simulated) solar light condition.

#### 3.4.6. PAMAM Dendrimers

PAMAM dendrimers [88] have a strong potential as a nanoplatform for OP sequestration. They offer various structural and functional benefits through their dendritic architecture, hydrophobic cavities and chemical stability [152,153,154]. Their applications in OP sequestration is demonstrated with a fourth generation (G4) PAMAM dendrimer functionalized with (L)-asparagine [155]. This amino acid-functionalized dendrimer has shown ability for rapid desorption and sequestration of hydrophobic OPs such as methamidophos and azinophos methyl.

**Figure 9:**
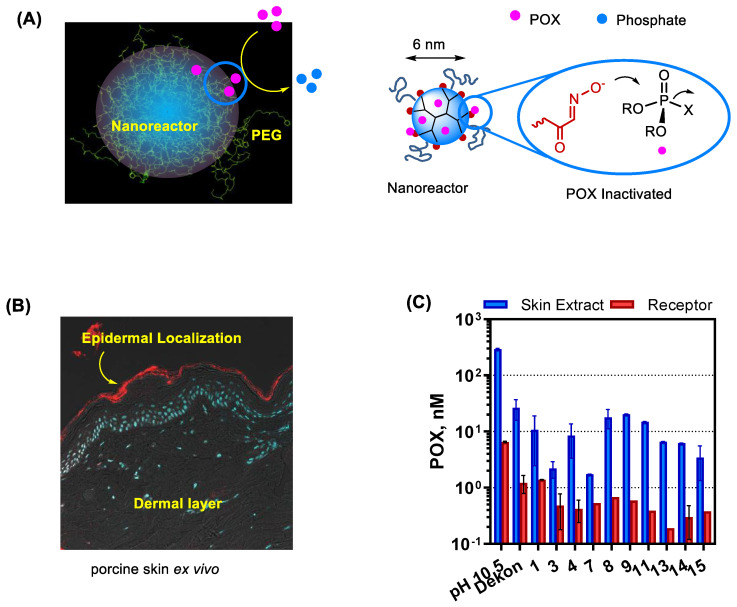
G5 PAMAM dendrimer-cored nanoreactors developed for topical decontamination of organophosphate (OP). (**A**) A proposed model for shielded oxime nanoreactor (left) and its mode of action in POX inactivation (right). (**B**) A confocal fluorescent image of the cryosection from porcine skin treated with Cy5 dye-labeled dendrimer nanoreactor ex vivo. (**C**) Efficacy of POX decontamination by a series of shielded oxime nanoreactors screened in porcine skin ex vivo. Reproduced with permission from [37], Copyright 2020, American Chemical Society.

PAMAM dendrimers can also serve a promising nanoreactor platform. They allow structural modifications with OP-reactive moieties on its peripheral branches as illustrated in Figure 8B. In this work, Bharathi et al. describes PAM-conjugated G5 dendrimer and its ability to accelerate the rate of POX hydrolysis [130]. However, despite such effectiveness, their OP-reactive moieties may engage in undesired reactions with proteins and enzymes such as AChE. To address such non-selectivity issue, Wong et al. [37] advanced its design concept to a shielded oxime nanoreactor as depicted in a model (Figure 9A). Its primary design strategy involves reactor shielding through partial surface PEGylation. Thus, the PEG layer can limit reactor accessibility to only small OPs via steric interference. Such PEG-shielded nanoreactors have shown rapid POX hydrolysis and effective POX decontamination in skin models ex vivo while they show lack of undesired effects such as AChE inhibition and skin penetration (Figure 9).

In topic summary, nanomaterials offer a variety of structural and functional properties that enable to address current unmet needs associated with OP exposure and intoxication. They have shown practical utilities in a broad range of applications from antidote drug delivery [86,87,89], protective material design [148], environmental decontamination [139] to therapeutic skin decontamination [37,117,118,130,144,149] as summarized in Table 2 and Table S1 (Supplementary Material).

## 4. Conclusions and Perspective

Over recent decades, nanomaterials have made a significant impact on advancing therapeutic and biomedical developments in a broad range of applications from therapeutics [20,21,22,23,24,25,26,27], imaging [156,157], diagnostics [158] to sensors [28]. This also applies in the field of OP exposure where urgent needs remain for its sensitive detection and efficacious treatment. This article has focused on this specific topic and discussed the status and prospect of nanomaterial applications that relate to OP detection, antidote drug delivery, and decontamination.

Nanosensors for OP analysis involve instrumental analysis via electrochemistry [43,44,45,46,47,48,49,50,51,52,53,54] or optical spectroscopy via absorption [59], fluorescence [55,56,57] or luminescence [58,61]. Compared to conventional sensors, these nanosensors have several advantages that include unique sensing mechanisms, greater loading capacity, higher sensitivity and faster onset of action. Some of these are attributable to intrinsic nanomaterial properties such as porosity [49], electric conductance [50,51,52,53,54], surface plasmon resonance (SPR) excitation [47,48,59], fluorescence [55,56,57] or luminescence [58,61] whereas others are due to a combination of chemical and biochemical functionalization made internally or externally with an aim to generate OP-specific signals in response to OP adsorption [51,52,53,56,57], reaction [54] or hydrolysis [47,48,49,55,60,61]. It is also of interest to note the role of magnetic control in MNP-based nanosensors, which offers convenience, on-off reversibility and high spatial precision in sensor assembly [43,44,45,46].

Nanomaterials serve as important platforms for OP treatment by engaging in either controlled drug delivery or OP sequestration (Table 2). Nanomaterials that contain internal cavities or pores are preferred as delivery platforms due to their loading capacity. This is evident with PAMAM dendrimer [86,87], human serum albumin [90], MOF [85] and SLN [84,91], each demonstrated for controlled drug release [86,87,89] or CNS delivery [84,91] of OP antidotes such as oxime drugs [84,85,86,87,90,91] or atropine [89]. Besides, they allow OP sequestration within their pores as demonstrated with MSN [144] and MOF [73].

Nanomaterials are readily functionalized to create mechanisms for OP inactivation by which they can serve as OP scavengers [159]. This is illustrated with those nanoscavengers made of liposome [120,134,139], polymer [140,141], MOF [117,148], metal oxide [149,150,151] and dendrimer [37,130,155]. Their scavenging capability relies on reactive functionalities either present in unmodified nanomaterials such as photocatalytic metal oxides [149,150,151] or built additionally such as oximes [120,134], metal chelates [140,141], amines [148] or amino acids [155]. Alternatively, specific functionalization is achieved through bioscavenger encapsulation such as phosphotriesterase [139] or OPAA [117] encapsulated in liposomes [139] or porous MOF [117]. Such nanoscavengers offer OP-targeted reactivity with lack of undesired percutaneous penetration due to their nm size, high polarity and charges [37,160]. Overall, such nanomaterial-enabled (bio)scavengers are validated as emerging therapeutic agents that could play an important role in topical OP decontamination.

## Figures and Tables

**Table 1:** Nanomaterial-enabled sensors developed for OP analysis.

Detection	Concept	Design	OP Analyte (LOD)	Ref
Electrochemistry	AChE Inhibition	IONP@AChE	Chlorpyrifos oxon, malathion (0.3 nM)	[43,45,46]
		nano Fe-Ni@AChE	Phosmet (0.1 nM)	[44]
		AuNP-CaCO_3_@AChE	Malathion, chlorpyrifos (0.1 nM)	[47]
		nano Ag@Chitosan-AChE	POX (15 nM)	[48]
		MSN@AChE	Dimethoate (6.5 nM)	[49]
	Anti-OP Antibody	GNS@Anti-parathion Ab	Parathion (0.2 fM)	[50]
	OP Adsorption	rGO@Cu	Parathion, fenitrothion, malathion (3 nM)	[51]
		rGO@AuNP-polymer	Malathion (0.1 nM)	[52]
		GNS@AuNP	Parathion methyl (2 nM)	[53]
	OP Reaction	GO@AuNP-acetophenone oxime	Diethyl cyanophosphonate, dimethoate, fenitrothion	[54]
Fluorescence (Luminescence) Spectroscopy	AChE Inhibition	Cd-Te QD	Paraoxon, GB, VX (0.1–8.0 nM)	[55]
	OP Adsorption	CdTe QD	Chlorpyrifos (0.1 nM)	[56]
		ZnS-Mn QD	Diethyl phosphorothioate	[57]
		Hf-doped MOF	Methylphosphonate	[58]
		AuNP@Rhodamine	Ethoprophos (37 nM)	[59]
	OP Reaction	CdS QD + Eosin Y	Chlorpyrifos (29 nM)	[60]
		UCN@Oxime probe	Dimethoate (0.14 μM)	[61]
Colorimetry & Spectrophotometry	AChE Inhibition	AuNR + AChE	Dichlorvos (45 fM)	[62]
	OP Adsorption	AuNP, AgNP	Ethion, parathion	[63]
		AuNP@Rhodamine	Ethoprophos (37 nM)	[59]
		Nano Ag@PVP	Chlorpyrifos (14 nM)	[64]

AuNP = gold nanoparticle; GNS = graphene nanosheet; rGO = reduced graphene oxide; IONP = iron oxide nanoparticle; MNP = magnetic nanoparticle; MOF = metal-organic framework; MSN = mesoporous silica nanoparticle; PVP = polyvinylpyrrolidone; QD = quantum dot; UCN = upconversion nanoparticle.

**Table 2:** Nanomaterial-based therapeutic platforms for applications in antidote drug delivery or reactive organophosphate (OP) inactivation.

Nanomaterial	Design Feature	Tested OP	Function	Ref
Lipid based (micelle, liposome)	Oxime, HA presented	Fenitrothion	Catalytic inactivation	[120,134,135,136]
	Phosphotriesterase encapsulated	POX	Catalytic inactivation	[139]
	2-PAM encapsulated	POX	Rat brain delivery	[84,91]
Polymer	Cu (II)-bipyridyl chelated	Parathion methyl	Catalytic inactivation	[141]
	La (catecholate) chelated	POX, Nerve agents	Catalytic inactivation	[140]
MSN	Unmodified	Dichlorvos	Adsorption; inactivation	[144]
MOF	UiO-66	OP	Adsorption	[73]
	UiO-66-NH_2_	POX, VX, Soman	Catalytic inactivation	[146,147,148]
	OPAA immobilized	Soman	Catalytic inactivation	[117]
Metal Oxide	CeO_2_	POX	Catalytic inactivation	[149]
	TiO_2_	Parathion	Photocatalytic inactivation	[150]
	Ag-ZnO	Chlorpyrifos	Photocatalytic inactivation	[151]
PAMAM dendrimer	Amino acid conjugated	Azhinophos methyl	Adsorption	[155]
	Oxime/HA conjugated	POX, Malathion	Catalytic inactivation	[37,130]
	2-PAM, atropine encapsulated	-	Extended drug release	[86,87,89]

MOF = metal-organic framework; MSN = mesoporous silica nanoparticle; OPAA = organophosphorus acid anhydrolase; PAMAM = poly(amidoamine); Paraoxon = POX.

## Supplementary Materials

The following are available online at https://www.mdpi.com/2079-4991/11/1/224/s1, Table S1: A summary of antidote molecules, reactive organophosphates (OPs) and their inactivation by nanoscavengers.

## References

[1] Mercey G., Verdelet T., Renou J., Kliachyna M., Baati R., Nachon F., Jean L., Renard P.-Y. (2012). Reactivators of Acetylcholinesterase Inhibited by Organophosphorus Nerve Agents. Acc. Chem. Res..

[2] Heldman E., Ashani Y., Raveh L., Rachaman E.S. (1986). Sugar conjugates of pyridinium aldoximes as antidotes against organophosphate poisoning. Carbohydr. Res..

[3] Xiong S., Deng Y., Zhou Y., Gong D., Xu Y., Yang L., Chen H., Chen L., Song T., Luo A. (2018). Current progress in biosensors for organophosphorus pesticides based on enzyme functionalized nanostructures: A review. Anal. Methods.

[4] Sparling D.W., Sparling D.W. (2016). Chapter 5—Current Use Pesticides. Ecotoxicology Essentials.

[5] Casida J.E., Quistad G.B. (2004). Organophosphate Toxicology:  Safety Aspects of Nonacetylcholinesterase Secondary Targets. Chem. Res. Toxicol..

[6] D’Agostino J., Zhang H., Kenaan C., Hollenberg P.F. (2015). Mechanism-Based Inactivation of Human Cytochrome P450 2B6 by Chlorpyrifos. Chem. Res. Toxicol..

[7] Lin V.S., Volk R.F., DeLeon A.J., Anderson L.N., Purvine S.O., Shukla A.K., Bernstein H.C., Smith J.N., Wright A.T. (2020). Structure Dependent Determination of Organophosphate Targets in Mammalian Tissues Using Activity-Based Protein Profiling. Chem. Res. Toxicol..

[8] Schopfer L.M., Voelker T., Bartels C.F., Thompson C.M., Lockridge O. (2005). Reaction Kinetics of Biotinylated Organophosphorus Toxicant, FP-biotin, with Human Acetylcholinesterase and Human Butyrylcholinesterase. Chem. Res. Toxicol..

[9] Guo J.-X., Wu J.J.Q., Wright J.B., Lushington G.H. (2006). Mechanistic Insight into Acetylcholinesterase Inhibition and Acute Toxicity of Organophosphorus Compounds:  A Molecular Modeling Study. Chem. Res. Toxicol..

[10] Franklin M.C., Rudolph M.J., Ginter C., Cassidy M.S., Cheung J. (2016). Structures of Paraoxon-inhibited Human Acetylcholinesterase Reveal Perturbations of the Acyl Loop and the Dimer Interface. Proteins Struct. Funct. Bioinform..

[11] Chambers H.W., Chambers J.E. (1989). An investigation of acetylcholinesterase inhibition and aging and choline acetyltransferase activity following a high level acute exposure to paraoxon. Pestic. Biochem. Physiol..

[12] Bajgar J., Gregory S.M. (2004). Organophosphates/Nerve Agent Poisoning: Mechanism of Action, Diagnosis, Prophylaxis, and Treatment. Advances in Clinical Chemistry.

[13] Kim K.-H., Kabir E., Jahan S.A. (2017). Exposure to pesticides and the associated human health effects. Sci. Total Environ..

[14] Sanson B., Nachon F., Colletier J.-P., Froment M.-T., Toker L., Greenblatt H.M., Sussman J.L., Ashani Y., Masson P., Silman I. (2009). Crystallographic Snapshots of Nonaged and Aged Conjugates of Soman with Acetylcholinesterase, and of a Ternary Complex of the Aged Conjugate with Pralidoxime. J. Med. Chem..

[15] Peter J.V., Sudarsan T.I., Moran J.L. (2014). Clinical features of organophosphate poisoning: A review of different classification systems and approaches. Indian J. Crit. Care Med..

[16] Wong P., Bhattacharjee S., Cannon J., Tang S., Yang K., Bowden S., Varnau V., O′Konek J.J., Choi S.K. (2019). Reactivity and Mechanism of α-Nucleophile Scaffolds as Catalytic Organophosphate Scavengers. Org. Biomol. Chem..

[17] Clarke J.F., Cordery S.F., Morgan N.A., Knowles P.K., Guy R.H. (2018). Dermal Absorption of Pesticide Residues. Chem. Res. Toxicol..

[18] Fryer A.D., Lein P.J., Howard A.S., Yost B.L., Beckles R.A., Jett D.A. (2004). Mechanisms of organophosphate insecticide-induced airway hyperreactivity. Am. J. Physiol. Lung Cell. Mol. Physiol..

[19] Ragnarsdottir K.V. (2000). Environmental fate and toxicology of organophosphate pesticides. J. Geol. Soc..

[20] Farokhzad O.C., Langer R. (2009). Impact of Nanotechnology on Drug Delivery. ACS Nano.

[21] Wong P.T., Choi S.K. (2015). Mechanisms of Drug Release in Nanotherapeutic Delivery Systems. Chem. Rev..

[22] Kukowska-Latallo J.F., Candido K.A., Cao Z., Nigavekar S.S., Majoros I.J., Thomas T.P., Balogh L.P., Khan M.K., Baker J.R. (2005). Nanoparticle Targeting of Anticancer Drug Improves Therapeutic Response in Animal Model of Human Epithelial Cancer. Cancer Res..

[23] Wong P.T., Chen D., Tang S., Yanik S., Payne M., Mukherjee J., Coulter A., Tang K., Tao K., Sun K. (2015). Modular Integration of Upconversion Nanocrystal-Dendrimer Composites for Folate Receptor-Specific Near Infrared Imaging and Light Triggered Drug Release. Small.

[24] Agasti S.S., Rana S., Park M.-H., Kim C.K., You C.-C., Rotello V.M. (2010). Nanoparticles for detection and diagnosis. Adv. Drug Delivery Rev..

[25] Biju V. (2014). Chemical modifications and bioconjugate reactions of nanomaterials for sensing, imaging, drug delivery and therapy. Chem. Soc. Rev..

[26] Choi S.K. (2020). Photoactivation Strategies for Therapeutic Release in Nanodelivery Systems. Adv. Ther..

[27] Choi S.K. (2020). Activation Strategies in Image-Guided Nanotherapeutic Delivery. J. Nanotheranostics.

[28] Stewart M.E., Anderton C.R., Thompson L.B., Maria J., Gray S.K., Rogers J.A., Nuzzo R.G. (2008). Nanostructured Plasmonic Sensors. Chem. Rev..

[29] Mu Q., Jiang G., Chen L., Zhou H., Fourches D., Tropsha A., Yan B. (2014). Chemical Basis of Interactions Between Engineered Nanoparticles and Biological Systems. Chem. Rev..

[30] Zhang Y., Bai Y., Jia J., Gao N., Li Y., Zhang R., Jiang G., Yan B. (2014). Perturbation of physiological systems by nanoparticles. Chem. Soc. Rev..

[31] Dahl M., Liu Y., Yin Y. (2014). Composite Titanium Dioxide Nanomaterials. Chem. Rev..

[32] Daniel M.-C., Astruc D. (2004). Gold Nanoparticles; Assembly, Supramolecular Chemistry, Quantum-Size-Related Properties, and Applications toward Biology, Catalysis, and Nanotechnology. Chem. Rev..

[33] Eder D. (2010). Carbon Nanotube−Inorganic Hybrids. Chem. Rev..

[34] Chen G., Qiu H., Prasad P.N., Chen X. (2014). Upconversion Nanoparticles: Design, Nanochemistry, and Applications in Theranostics. Chem. Rev..

[35] Haase M., Schäfer H. (2011). Upconverting Nanoparticles. Angew. Chem. Int. Ed..

[36] Xia Y., Li W., Cobley C.M., Chen J., Xia X., Zhang Q., Yang M., Cho E.C., Brown P.K. (2011). Gold Nanocages: From Synthesis to Theranostic Applications. Acc. Chem. Res..

[37] Wong P.T., Tang S., Cannon J., Yang K., Harrison R., Ruge M., O’Konek J.J., Choi S.K. (2020). Shielded α-Nucleophile Nanoreactor for Topical Decontamination of Reactive Organophosphate. ACS Appl. Mater. Interfaces.

[38] Pang Z., Hu C.-M.J., Fang R.H., Luk B.T., Gao W., Wang F., Chuluun E., Angsantikul P., Thamphiwatana S., Lu W. (2015). Detoxification of Organophosphate Poisoning Using Nanoparticle Bioscavengers. ACS Nano.

[39] Ju H., Kandimalla V.B., Zhang X., Ju H., Wang J. (2008). CHAPTER 2—Biosensors for pesticides. Electrochemical Sensors, Biosensors and Their Biomedical Applications.

[40] Alonso G.A., Muñoz R., Marty J.-L. (2013). Automatic Electronic Tongue for On-Line Detection and Quantification of Organophosphorus and Carbamate Pesticides Using Enzymatic Screen Printed Biosensors. Anal. Lett..

[41] Cortina M., del Valle M., Marty J.-L. (2008). Electronic Tongue Using an Enzyme Inhibition Biosensor Array for the Resolution of Pesticide Mixtures. Electroanalysis.

[42] Liu X., Song M., Hou T., Li F. (2017). Label-Free Homogeneous Electroanalytical Platform for Pesticide Detection Based on Acetylcholinesterase-Mediated DNA Conformational Switch Integrated with Rolling Circle Amplification. ACS Sens..

[43] Rodrigues N.F.M., Neto S.Y., Luz R.D.C.S., Damos F.S., Yamanaka H. (2018). Ultrasensitive Determination of Malathion Using Acetylcholinesterase Immobilized on Chitosan-Functionalized Magnetic Iron Nanoparticles. Biosensors.

[44] El-Moghazy A.Y., Soliman E.A., Ibrahim H.Z., Noguer T., Marty J.L., Istamboulie G. (2016). Ultra-sensitive biosensor based on genetically engineered acetylcholinesterase immobilized in poly (vinyl alcohol)/Fe–Ni alloy nanocomposite for phosmet detection in olive oil. Food Chem..

[45] Istamboulie G., Andreescu S., Marty J.-L., Noguer T. (2007). Highly sensitive detection of organophosphorus insecticides using magnetic microbeads and genetically engineered acetylcholinesterase. Biosens. Bioelectron..

[46] Dominguez R.B., Alonso G.A., Muñoz R., Hayat A., Marty J.-L. (2015). Design of a novel magnetic particles based electrochemical biosensor for organophosphate insecticide detection in flow injection analysis. Sens. Actuators B Chem..

[47] Chauhan N., Narang J., Pundir C.S. (2011). Immobilization of rat brain acetylcholinesterase on porous gold-nanoparticle–CaCO3 hybrid material modified Au electrode for detection of organophosphorous insecticides. Int. J. Biol. Macromol..

[48] Zheng Q., Yu Y., Fan K., Ji F., Wu J., Ying Y. (2016). A nano-silver enzyme electrode for organophosphorus pesticide detection. Anal. Bioanal. Chem..

[49] Palanivelu J., Chidambaram R. (2019). Acetylcholinesterase with Mesoporous Silica: Covalent Immobilization, Physiochemical Characterization, and Its Application in Food for Pesticide Detection. J. Cell. Biochem..

[50] Mehta J., Vinayak P., Tuteja S.K., Chhabra V.A., Bhardwaj N., Paul A.K., Kim K.-H., Deep A. (2016). Graphene modified screen printed immunosensor for highly sensitive detection of parathion. Biosens. Bioelectron..

[51] Li Z., Zhang H., Ge X., Liang Y., An X., Yang C., Fang B., Xie H., Wei J. (2013). A nanocomposite of copper(ii) functionalized graphene and application for sensing sulfurated organophosphorus pesticides. New J. Chem..

[52] Facure M.H.M., Mercante L.A., Mattoso L.H.C., Correa D.S. (2017). Detection of trace levels of organophosphate pesticides using an electronic tongue based on graphene hybrid nanocomposites. Talanta.

[53] Gong J., Miao X., Zhou T., Zhang L. (2011). An enzymeless organophosphate pesticide sensor using Au nanoparticle-decorated graphene hybrid nanosheet as solid-phase extraction. Talanta.

[54] Huixiang W., Danqun H., Yanan Z., Na M., Jingzhou H., Miao L., Caihong S., Changjun H. (2017). A non-enzymatic electro-chemical sensor for organophosphorus nerve agents mimics and pesticides detection. Sens. Actuators B Chem..

[55] Yu T., Ying T.-Y., Song Y.-Y., Li Y.-J., Wu F.-H., Dong X.-Q., Shen J.-S. (2014). A highly sensitive sensing system based on photoluminescent quantum dots for highly toxic organophosphorus compounds. RSC Adv..

[56] Zhang K., Mei Q., Guan G., Liu B., Wang S., Zhang Z. (2010). Ligand Replacement-Induced Fluorescence Switch of Quantum Dots for Ultrasensitive Detection of Organophosphorothioate Pesticides. Anal. Chem..

[57] Zhang K., Yu T., Liu F., Sun M., Yu H., Liu B., Zhang Z., Jiang H., Wang S. (2014). Selective Fluorescence Turn-On and Ratiometric Detection of Organophosphate Using Dual-Emitting Mn-Doped ZnS Nanocrystal Probe. Anal. Chem..

[58] Lian X., Yan B. (2018). Trace Detection of Organophosphorus Chemical Warfare Agents in Wastewater and Plants by Luminescent UIO-67(Hf) and Evaluating the Bioaccumulation of Organophosphorus Chemical Warfare Agents. ACS Appl. Mater. Interfaces.

[59] Li X., Cui H., Zeng Z. (2018). A Simple Colorimetric and Fluorescent Sensor to Detect Organophosphate Pesticides Based on Adenosine Triphosphate-Modified Gold Nanoparticles. Sensors.

[60] Dey P.C., Das R. (2019). Ligand free surface of CdS nanoparticles enhances the energy transfer efficiency on interacting with Eosin Y dye—Helping in the sensing of very low level of chlorpyrifos in water. Spectrochim. Acta Part A.

[61] Wang S., Wang X., Chen X., Cao X., Cao J., Xiong X., Zeng W. (2016). A novel upconversion luminescence turn-on nanosensor for ratiometric detection of organophosphorus pesticides. RSC Adv..

[62] Liu Y., Lv B., Liu A., Liang G., Yin L., Pu Y., Wei W., Gou S., Liu S. (2018). Multicolor sensor for organophosphorus pesticides determination based on the bi-enzyme catalytic etching of gold nanorods. Sens. Actuators B Chem..

[63] Dissanayake N.M., Arachchilage J.S., Samuels T.A., Obare S.O. (2019). Highly sensitive plasmonic metal nanoparticle-based sensors for the detection of organophosphorus pesticides. Talanta.

[64] Sarkar S., Das R. (2018). Presence of chlorpyrifos shows blue shift of the absorption peak of silver nanohexagons solution—An indication of etching of nanocrystals and sensing of chlorpyrifos. Sens. Actuators B Chem..

[65] Chen Y., Tan C., Zhang H., Wang L. (2015). Two-dimensional graphene analogues for biomedical applications. Chem. Soc. Rev..

[66] Paliwal S., Wales M., Good T., Grimsley J., Wild J., Simonian A. (2007). Fluorescence-based sensing of p-nitrophenol and p-nitrophenyl substituent organophosphates. Anal. Chim. Acta.

[67] Obare S.O., De C., Guo W., Haywood T.L., Samuels T.A., Adams C.P., Masika N.O., Murray D.H., Anderson G.A., Campbell K. (2010). Fluorescent Chemosensors for Toxic Organophosphorus Pesticides: A Review. Sensors.

[68] Zhou J., Yang Y., Zhang C.-y. (2015). Toward Biocompatible Semiconductor Quantum Dots: From Biosynthesis and Bioconjugation to Biomedical Application. Chem. Rev..

[69] Idris N.M., Jayakumar M.K.G., Bansal A., Zhang Y. (2015). Upconversion nanoparticles as versatile light nanotransducers for photoactivation applications. Chem. Soc. Rev..

[70] Heer S., Kömpe K., Güdel H.U., Haase M. (2004). Highly Efficient Multicolour Upconversion Emission in Transparent Colloids of Lanthanide-Doped NaYF4 Nanocrystals. Adv. Mater..

[71] Tao K., Sun K., Choi S.K., Choi S.K. (2020). Chapter 12—Upconversion nanocrystals for near-infrared-controlled drug delivery. Photonanotechnology for Therapeutics and Imaging.

[72] Vikrant K., Tsang D.C.W., Raza N., Giri B.S., Kukkar D., Kim K.-H. (2018). Potential Utility of Metal–Organic Framework-Based Platform for Sensing Pesticides. ACS Appl. Mater. Interfaces.

[73] Dhaka S., Kumar R., Deep A., Kurade M.B., Ji S.-W., Jeon B.-H. (2019). Metal–organic frameworks (MOFs) for the removal of emerging contaminants from aquatic environments. Coord. Chem. Rev..

[74] Yan B. (2017). Lanthanide-Functionalized Metal–Organic Framework Hybrid Systems To Create Multiple Luminescent Centers for Chemical Sensing. Acc. Chem. Res..

[75] Singha D.K., Majee P., Mondal S.K., Mahata P. (2017). Highly Selective Aqueous Phase Detection of Azinphos-Methyl Pesticide in ppb Level Using a Cage-Connected 3D MOF. ChemistrySelect.

[76] Tang J., Ma X., Yang J., Feng D.-D., Wang X.-Q. (2020). Recent advances in metal–organic frameworks for pesticide detection and adsorption. Dalton Trans..

[77] Chen H., Shao L., Li Q., Wang J. (2013). Gold nanorods and their plasmonic properties. Chem. Soc. Rev..

[78] Marrs T.C., Rice P., Vale J.A. (2006). The Role of Oximes in the Treatment of Nerve Agent Poisoning in Civilian Casualties. Toxicol. Rev..

[79] Snider T.H., Wilhelm C.M., Babin M.C., Platoff G.E., Yeung D.T. (2015). Assessing the therapeutic efficacy of oxime therapies against percutaneous organophosphorus pesticide and nerve agent challenges in the Hartley guinea pig. J. Toxicol. Sci..

[80] Reddy S.D., Reddy D.S. (2015). Midazolam as an anticonvulsant antidote for organophosphate intoxication—A pharmacotherapeutic appraisal. Epilepsia.

[81] Sit R.K., Radić Z., Gerardi V., Zhang L., Garcia E., Katalinić M., Amitai G., Kovarik Z., Fokin V.V., Sharpless K.B. (2011). New Structural Scaffolds for Centrally Acting Oxime Reactivators of Phosphylated Cholinesterases. J. Biol. Chem..

[82] DeMar J.C., Clarkson E.D., Ratcliffe R.H., Campbell A.J., Thangavelu S.G., Herdman C.A., Leader H., Schulz S.M., Marek E., Medynets M.A. (2010). Pro-2-PAM Therapy for Central and Peripheral Cholinesterases. Chem.-Biol. Interact..

[83] Jovanović D. (1989). Pharmacokinetics of Pralidoxime Chloride. Arch. Toxicol..

[84] Pashirova T.N., Braïki A., Zueva I.V., Petrov K.A., Babaev V.M., Burilova E.A., Samarkina D.A., Rizvanov I.K., Souto E.B., Jean L. (2018). Combination delivery of two oxime-loaded lipid nanoparticles: Time-dependent additive action for prolonged rat brain protection. J. Control. Release.

[85] Vilela S.M.F., Salcedo-Abraira P., Colinet I., Salles F., De Koning M.C., Joosen M.J.A., Serre C., Horcajada P. (2017). Nanometric MIL-125-NH2 Metal–Organic Framework as a Potential Nerve Agent Antidote Carrier. Nanomaterials.

[86] Choi S.K., Thomas T.P., Leroueil P.R., Kotlyar A., Van Der Spek A.F.L., Baker J.R. (2012). Specific and Cooperative Interactions between Oximes and PAMAM Dendrimers as Demonstrated by 1H NMR Study. J. Phys. Chem. B.

[87] Choi S.K., Leroueil P., Li M.-H., Desai A., Zong H., Van Der Spek A.F.L., Baker J.R. (2011). Specificity and Negative Cooperativity in Dendrimer–Oxime Drug Complexation. Macromolecules.

[88] Tomalia D.A., Naylor A.M., William A., Goddard I. (1990). Starburst Dendrimers: Molecular-Level Control of Size, Shape, Surface Chemistry, Topology, and Flexibility from Atoms to Macroscopic Matter. Angew. Chem. Int. Ed..

[89] Mukherjee J., Wong P.T., Tang S., Gam K., Coulter A., Baker J.R., Choi S.K. (2015). Mechanism of Cooperativity and Nonlinear Release Kinetics in Multivalent Dendrimer-Atropine Complexes. Mol. Pharm..

[90] Wagner S., Kufleitner J., Zensi A., Dadparvar M., Wien S., Bungert J., Vogel T., Worek F., Kreuter J., von Briesen H. (2010). Nanoparticulate Transport of Oximes over an In Vitro Blood-Brain Barrier Model. PLoS ONE.

[91] Pashirova T.N., Zueva I.V., Petrov K.A., Babaev V.M., Lukashenko S.S., Rizvanov I.K., Souto E.B., Nikolsky E.E., Zakharova L.Y., Masson P. (2017). Nanoparticle-Delivered 2-PAM for Rat Brain Protection against Paraoxon Central Toxicity. ACS Appl. Mater. Interfaces.

[92] Hinderling P.H., Gundert-Remy U., Schmidlin O. (1985). Integrated pharmacokinetics and pharmacodynamics of atropine in healthy humans I: Pharmacokinetics. J. Pharm. Sci..

[93] Han J., Lim S.-J., Lee M.-K., Kim C.-K. (2001). Altered Pharmacokinetics and Liver Targetability of Methotrexate by Conjugation with Lactosylated Albumins. Drug Deliv..

[94] Morgan M.T., Nakanishi Y., Kroll D.J., Griset A.P., Carnahan M.A., Wathier M., Oberlies N.H., Manikumar G., Wani M.C., Grinstaff M.W. (2006). Dendrimer-Encapsulated Camptothecins: Increased Solubility, Cellular Uptake, and Cellular Retention Affords Enhanced Anticancer Activity In vitro. Cancer Res..

[95] He Y., Zeng S., Abd El-Aty A.M., Hacımüftüoğlu A., Kalekristos Yohannes W., Khan M., She Y. (2020). Development of Water-Compatible Molecularly Imprinted Polymers Based on Functionalized β-Cyclodextrin for Controlled Release of Atropine. Polymers.

[96] Jacquet P., Daudé D., Bzdrenga J., Masson P., Elias M., Chabrière E. (2016). Current and emerging strategies for organophosphate decontamination: Special focus on hyperstable enzymes. Environ. Sci. Pollut. Res..

[97] Goldsmith M., Ashani Y. (2018). Catalytic bioscavengers as countermeasures against organophosphate nerve agents. Chem.-Biol. Interact..

[98] Iyengar A.R.S., Pande A.H. (2016). Organophosphate-Hydrolyzing Enzymes as First-Line of Defence Against Nerve Agent-Poisoning: Perspectives and the Road Ahead. Protein J..

[99] Schenk G., Mateen I., Ng T.-K., Pedroso M.M., Mitić N., Jafelicci M., Marques R.F.C., Gahan L.R., Ollis D.L. (2016). Organophosphate-degrading metallohydrolases: Structure and function of potent catalysts for applications in bioremediation. Coord. Chem. Rev..

[100] Radić Z., Dale T., Kovarik Z., Berend S., Garcia E., Zhang L., Amitai G., Green C., Radić B., Duggan B.M. (2013). Catalytic detoxification of nerve agent and pesticide organophosphates by butyrylcholinesterase assisted with non-pyridinium oximes. Biochem. J..

[101] Sit R.K., Fokin V.V., Amitai G., Sharpless K.B., Taylor P., Radić Z. (2014). Imidazole Aldoximes Effective in Assisting Butyrylcholinesterase Catalysis of Organophosphate Detoxification. J. Med. Chem..

[102] Huang Y.-J., Huang Y., Baldassarre H., Wang B., Lazaris A., Leduc M., Bilodeau A.S., Bellemare A., Côté M., Herskovits P. (2007). Recombinant human butyrylcholinesterase from milk of transgenic animals to protect against organophosphate poisoning. Proc. Natl. Acad. Sci. USA.

[103] Hemmert A.C., Otto T.C., Wierdl M., Edwards C.C., Fleming C.D., MacDonald M., Cashman J.R., Potter P.M., Cerasoli D.M., Redinbo M.R. (2010). Human Carboxylesterase 1 Stereoselectively Binds the Nerve Agent Cyclosarin and Spontaneously Hydrolyzes the Nerve Agent Sarin. Mol. Pharmacol..

[104] Lenz D.E., Yeung D., Smith J.R., Sweeney R.E., Lumley L.A., Cerasoli D.M. (2007). Stoichiometric and catalytic scavengers as protection against nerve agent toxicity: A mini review. Toxicology.

[105] Valiyaveettil M., Alamneh Y., Rezk P., Biggemann L., Perkins M.W., Sciuto A.M., Doctor B.P., Nambiar M.P. (2011). Protective efficacy of catalytic bioscavenger, paraoxonase 1 against sarin and soman exposure in guinea pigs. Biochem. Pharmacol..

[106] Zhang P., Liu E.J., Tsao C., Kasten S.A., Boeri M.V., Dao T.L., DeBus S.J., Cadieux C.L., Baker C.A., Otto T.C. (2019). Nanoscavenger provides long-term prophylactic protection against nerve agents in rodents. Sci. Transl. Med..

[107] Sogorb M.A., García-Argüelles S., Carrera V., Vilanova E. (2008). Serum Albumin is as Efficient as Paraxonase in the Detoxication of Paraoxon at Toxicologically Relevant Concentrations. Chem. Res. Toxicol..

[108] Cohen O., Kronman C., Raveh L., Mazor O., Ordentlich A., Shafferman A. (2006). Comparison of Polyethylene Glycol-Conjugated Recombinant Human Acetylcholinesterase and Serum Human Butyrylcholinesterase as Bioscavengers of Organophosphate Compounds. Mol. Pharmacol..

[109] Jun D., Musilová L., Link M., Loiodice M., Nachon F., Rochu D., Renault F., Masson P. (2010). Preparation and Characterization of Methoxy Polyethylene Glycol-conjugated Phosphotriesterase As a Potential Catalytic Bioscavenger against Organophosphate Poisoning. Chem.-Biol. Interact..

[110] Worek F., Thiermann H., Wille T. (2016). Oximes in Organophosphate Poisoning: 60 Years of Hope and Despair. Chem.-Biol. Interact..

[111] Kovarik Z., Maček Hrvat N., Katalinić M., Sit R.K., Paradyse A., Žunec S., Musilek K., Fokin V.V., Taylor P., Radić Z. (2015). Catalytic Soman Scavenging by the Y337A/F338A Acetylcholinesterase Mutant Assisted with Novel Site-Directed Aldoximes. Chem. Res. Toxicol..

[112] Trovaslet-Leroy M., Musilova L., Renault F., Brazzolotto X., Misik J., Novotny L., Froment M.-T., Gillon E., Loiodice M., Verdier L. (2011). Organophosphate hydrolases as catalytic bioscavengers of organophosphorus nerve agents. Toxicol. Lett..

[113] Kronman C., Cohen O., Raveh L., Mazor O., Ordentlich A., Shafferman A. (2007). Polyethylene-glycol conjugated recombinant human acetylcholinesterase serves as an efficacious bioscavenger against soman intoxication. Toxicology.

[114] Noy-Porat T., Cohen O., Ehrlich S., Epstein E., Alcalay R., Mazor O. (2015). Acetylcholinesterase-Fc Fusion Protein (AChE-Fc): A Novel Potential Organophosphate Bioscavenger with Extended Plasma Half-Life. Bioconjug. Chem..

[115] Misik J., Pavlikova R., Josse D., Cabal J., Kuca K. (2012). In vitro skin permeation and decontamination of the organophosphorus pesticide paraoxon under various physical conditions—Evidence for a wash-in effect. Toxicol. Mech. Methods.

[116] Bjarnason S., Mikler J., Hill I., Tenn C., Garrett M., Caddy N., Sawyer T. (2008). Comparison of Selected Skin Decontaminant Products and Regimens Against VX In Domestic Swine. Hum. Exp. Toxicol..

[117] Li P., Moon S.-Y., Guelta M.A., Lin L., Gómez-Gualdrón D.A., Snurr R.Q., Harvey S.P., Hupp J.T., Farha O.K. (2016). Nanosizing a Metal–Organic Framework Enzyme Carrier for Accelerating Nerve Agent Hydrolysis. ACS Nano.

[118] Amitai G., Murata H., Andersen J.D., Koepsel R.R., Russell A.J. (2010). Decontamination of Chemical and Biological Warfare Agents with a Single Multi-functional Material. Biomaterials.

[119] Morales J.I., Figueroa R., Rojas M., Millán D., Tapia R.A., Pavez P. (2018). Dual function of amino acid ionic liquids (Bmim[AA]) on the degradation of the organophosphorus pesticide, Paraoxon^®^. Org. Biomol. Chem..

[120] Singh N., Karpichev Y., Sharma R., Gupta B., Sahu A.K., Satnami M.L., Ghosh K.K. (2015). From α-nucleophiles to functionalized aggregates: Exploring the reactivity of hydroxamate ion towards esterolytic reactions in micelles. Org. Biomol. Chem..

[121] Tsang J.S.W., Neverov A.A., Brown R.S. (2004). La^3+^-catalyzed methanolysis of O,O-diethyl S-(p-nitrophenyl) phosphorothioate and O,O-diethyl S-phenyl phosphorothioate. Millions-fold acceleration of the destruction of V-agent simulants. Org. Biomol. Chem..

[122] Braue E.H., Smith K.H., Doxzon B.F., Lumpkin H.L., Clarkson E.D. (2011). Efficacy Studies of Reactive Skin Decontamination Lotion, M291 Skin Decontamination Kit, 0.5% Bleach, 1% Soapy Water, and Skin Exposure Reduction Paste Against Chemical Warfare Agents, Part 1: Guinea Pigs Challenged with VX. Cutan. Ocul. Toxicol..

[123] Fentabil M., Gebremedhin M., Purdon J.G., Cochrane L., Goldman V.S. (2018). Degradation of Pesticides with RSDL® (Reactive Skin Decontamination Lotion Kit) Lotion: LC–MS Investigation. Toxicol. Lett..

[124] Tang S., Wong P.T., Cannon J., Yang K., Bowden S., Bhattacharjee S., O’Konek J.J., Choi S.K. (2019). Hydrophilic Scaffolds of Oxime as the Potent Catalytic Inactivator of Reactive Organophosphate. Chem.-Biol. Interact..

[125] Fryer M.W., Gage P.W., Neering I.R., Dulhunty A.F., Lamh G.D. (1988). Paralysis of skeletal muscle by butanedione monoxime, a chemical phosphatase. Pflugers Arch-Eur. J. Physiol..

[126] Um I.-H., Jeon S.-E., Baek M.-H., Park H.-R. (2003). Significant and differential acceleration of dephosphorylation of the insecticides, paraoxon and parathion, caused by alkali metal ethoxides. Chem. Commun..

[127] Orth E.S., Almeida T.G., Silva V.B., Oliveira A.R.M., Ocampos F.M.M., Barison A. (2015). Mechanistic insight on the catalytic detoxification of Paraoxon mediated by imidazole: Furnishing optimum scaffolds for scavenging organophosphorus agents. J. Mol. Catal. A Chem..

[128] Wilson C., Cooper N.J., Briggs M.E., Cooper A.I., Adams D.J. (2018). Investigating the breakdown of the nerve agent simulant methyl paraoxon and chemical warfare agents GB and VX using nitrogen containing bases. Org. Biomol. Chem..

[129] Terrier F., Rodriguez-Dafonte P., Le Guevel E., Moutiers G. (2006). Revisiting the Reactivity of Oximate a-Nucleophiles with Electrophilic Phosphorus Centers. Relevance to Detoxification of Sarin, Soman and DFP Under Mild Conditions. Org. Biomol. Chem..

[130] Bharathi S., Wong P.T., Desai A., Lykhytska O., Choe V., Kim H., Thomas T.P., Baker J.R., Choi S.K. (2014). Design and Mechanistic Investigation of Oxime-conjugated PAMAM Dendrimers As the Catalytic Scavenger of Reactive Organophosphate. J. Mater. Chem. B.

[131] Behrman E.J., Biallas M.J., Brass H.J., Edwards J.O., Isaks M. (1970). Reactions of Phosphonic Acid Esters with Nucleophiles. II. Survey of Nucleophiles Reacting with p-Nitrophenyl Methylphosphonate Anion. J. Org. Chem..

[132] Le Provost R., Wille T., Louise L., Masurier N., Muller S., Reiter G., Renard P.-Y., Lafont O., Worek F., Estour F. (2011). Optimized strategies to synthesize b-cyclodextrin-oxime conjugates as a new generation of organophosphate scavengers. Org. Biomol. Chem..

[133] Masurier N., Estour F., Froment M.-T., Lefèvre B., Debouzy J.-C., Brasme B., Masson P., Lafont O. (2005). Synthesis of 2-substituted β-cyclodextrin derivatives with a hydrolytic activity against the organophosphorylester paraoxon. Eur. J. Med. Chem..

[134] Han X., Balakrishnan V.K., van Loon G.W., Buncel E. (2006). Degradation of the Pesticide Fenitrothion as Mediated by Cationic Surfactants and a-Nucleophilic Reagents. Langmuir.

[135] Kandpal N., Dewangan H.K., Nagwanshi R., Ghosh K.K., Satnami M.L. (2018). Micellar-accelerated hydrolysis of organophosphate and thiophosphates by pyridine oximate. Int. J. Chem. Kinet..

[136] Gonçalves L.M., Kobayakawa T.G., Zanette D., Chaimovich H., Cuccovia I.M. (2009). Effects of Micelles and Vesicles on the Oximolysis of p-Nitrophenyl Diphenyl Phosphate: A Model System for Surfactant-Based Skin-Defensive Formulations against Organophosphates. J. Pharm. Sci..

[137] Kapitanov I.V., Mirgorodskaya A.B., Valeeva F.G., Gathergood N., Kuca K., Zakharova L.Y., Karpichev Y. (2017). Physicochemical properties and esterolytic reactivity of oxime functionalized surfactants in pH-responsive mixed micellar system. Colloids Surf. A Physicochem. Eng. Asp..

[138] Kandpal N., Dewangan H.K., Nagwanshi R., Ghosh K.K., Satnami M.L. (2017). An investigation of kinetic and physicochemical properties of vesicular surfactants with oximate and hydroxamate ions: Hydrolytic reactions of organophosphorus pesticides. J. Mol. Liq..

[139] Alves N.J., Moore M., Johnson B.J., Dean S.N., Turner K.B., Medintz I.L., Walper S.A. (2018). Environmental Decontamination of a Chemical Warfare Simulant Utilizing a Membrane Vesicle-Encapsulated Phosphotriesterase. ACS Appl. Mater. Interfaces.

[140] Totten R.K., Weston M.H., Park J.K., Farha O.K., Hupp J.T., Nguyen S.T. (2013). Catalytic Solvolytic and Hydrolytic Degradation of Toxic Methyl Paraoxon with La(catecholate)-Functionalized Porous Organic Polymers. ACS Catal..

[141] Hartshorn C.M., Singh A., Chang E.L. (2002). Metal-chelator polymers as organophosphate hydrolysis catalysts. J. Mater. Chem..

[142] Wu S.-H., Mou C.-Y., Lin H.-P. (2013). Synthesis of mesoporous silica nanoparticles. Chem. Soc. Rev..

[143] Tarn D., Ashley C.E., Xue M., Carnes E.C., Zink J.I., Brinker C.J. (2013). Mesoporous Silica Nanoparticle Nanocarriers: Biofunctionality and Biocompatibility. Acc. Chem. Res..

[144] Xu P., Guo S., Yu H., Li X. (2014). Mesoporous Silica Nanoparticles (MSNs) for Detoxification of Hazardous Organophorous Chemicals. Small.

[145] Horcajada P., Gref R., Baati T., Allan P.K., Maurin G., Couvreur P., Férey G., Morris R.E., Serre C. (2012). Metal–Organic Frameworks in Biomedicine. Chem. Rev..

[146] Katz M.J., Moon S.-Y., Mondloch J.E., Beyzavi M.H., Stephenson C.J., Hupp J.T., Farha O.K. (2015). Exploiting parameter space in MOFs: A 20-fold enhancement of phosphate-ester hydrolysis with UiO-66-NH2. Chem. Sci..

[147] Katz M.J., Mondloch J.E., Totten R.K., Park J.K., Nguyen S.T., Farha O.K., Hupp J.T. (2014). Simple and Compelling Biomimetic Metal–Organic Framework Catalyst for the Degradation of Nerve Agent Simulants. Angew. Chem. Int. Ed..

[148] de Koning M.C., van Grol M., Breijaert T. (2017). Degradation of Paraoxon and the Chemical Warfare Agents VX, Tabun, and Soman by the Metal–Organic Frameworks UiO-66-NH2, MOF-808, NU-1000, and PCN-777. Inorg. Chem..

[149] Salerno A., Devers T., Bolzinger M.-A., Pelletier J., Josse D., Briançon S. (2017). In Vitro Skin Decontamination of the Organophosphorus Pesticide Paraoxon with Nanometric Cerium Oxide CeO_2_. Chem.-Biol. Interact..

[150] Konstantinou I.K., Sakellarides T.M., Sakkas V.A., Albanis T.A. (2001). Photocatalytic Degradation of Selected s-Triazine Herbicides and Organophosphorus Insecticides over Aqueous TiO_2_ Suspensions. Environ. Sci. Technol..

[151] Choudhary M.K., Kataria J., Bhardwaj V.K., Sharma S. (2019). Green biomimetic preparation of efficient Ag–ZnO heterojunctions with excellent photocatalytic performance under solar light irradiation: A novel biogenic-deposition-precipitation approach. Nanoscale Adv..

[152] Svenson S., Tomalia D.A. (2005). Dendrimers in Biomedical Applications--Reflections on the Field. Adv. Drug Deliv. Rev..

[153] Shcharbin D., Janaszewska A., Klajnert-Maculewicz B., Ziemba B., Dzmitruk V., Halets I., Loznikova S., Shcharbina N., Milowska K., Ionov M. (2014). How to Study Dendrimers and Dendriplexes III. Biodistribution, Pharmacokinetics and Toxicity In Vivo. J. Control. Release.

[154] Roberts J.C., Bhalgat M.K., Zera R.T. (1996). Preliminary Biological Evaluation of Polyamidoamine (PAMAM) StarburstTM Dendrimers. J. Biomed. Mater. Res..

[155] Durán-Lara E.F., Ávila-Salas F., Galaz S., John A., Maricán A., Gutiérrez M., Nachtigall F.M., Gonzalez-Nilo F.D., Santos L.S. (2015). Nano-Detoxification of Organophosphate Agents by PAMAM Derivatives. J. Braz. Chem. Soc..

[156] Shi X., Wang S.H., Swanson S.D., Ge S., Cao Z., Van Antwerp M.E., Landmark K.J., Baker J.R. (2008). Dendrimer-Functionalized Shell-crosslinked Iron Oxide Nanoparticles for In-Vivo Magnetic Resonance Imaging of Tumors. Adv. Mater..

[157] Lim E.-K., Kim T., Paik S., Haam S., Huh Y.-M., Lee K. (2015). Nanomaterials for Theranostics: Recent Advances and Future Challenges. Chem. Rev..

[158] Rosi N.L., Mirkin C.A. (2005). Nanostructures in Biodiagnostics. Chem. Rev..

[159] Aragay G., Pino F., Merkoçi A. (2012). Nanomaterials for Sensing and Destroying Pesticides. Chem. Rev..

[160] Yang Y., Sunoqrot S., Stowell C., Ji J., Lee C.-W., Kim J.W., Khan S.A., Hong S. (2012). Effect of Size, Surface Charge, and Hydrophobicity of Poly(amidoamine) Dendrimers on Their Skin Penetration. Biomacromolecules.

