# Supplementary material for: Nanomaterial-Enabled Sensors and Therapeutic Platforms for Reactive Organophosphates
Source: Nanomaterials (Basel). 2021 Jan 16;11(1):224. doi: 10.3390/nano11010224 (PMC7830340; doi:10.3390/nano11010224)
Supplement: Supplementary file 1 [file nanomaterials-11-00224-s001.pdf]

Supplementary Material

Nanomaterial-Enabled Sensors and Therapeutic Platforms for Reactive Organophosphates

Seok Ki Choi [1,2,*]

[1] Michigan Nanotechnology Institute for Medicine and Biological Sciences, University of Michigan Medical School, Ann Arbor, Michigan 48109, United States of America
[2] Department of Internal Medicine, University of Michigan Medical School, Ann Arbor, Michigan 48109, United States of America
* Correspondence: skchoi@umich.edu

Table S1. A summary of antidote molecules, reactive organophosphates (OPs) and their inactivation by nanoscavengers

**Antidotes and Topical Decontaminants**				
**Compound**	**Formula**	**Classification**	**Mode of Action**	**Delivery System**
2-PAM	$C_7H_9N_2O$	Antidote	AChE reactivation	PAMAM dendrimer [1, 2]; 2-PAM-encapsulated SLN [3, 4]
Atropine	$C_{17}H_{23}NO_3$	Antidote	Acetylcholine receptor antagonist (blocker)	PAMAM dendrimer [5]
Dekon 139	$C_4H_6KNO_2$	Topical Decontaminant	OP inactivation	RSDL [6, 7]
Oxime/HA scaffolds	-	Topical Decontaminant	OP inactivation	[8, 9]
**Reactive Organophosphates (Nerve Agents, Pesticides)**				
**OP**	**Formula**	**OP Inactivation (Degradation) and Byproducts**		**Nano (Bio)Scavenger**
Sarin (GB)	$C_4H_{10}FO_2P$	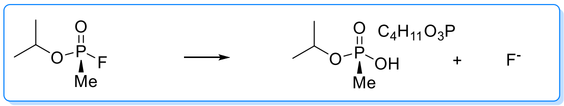		RSDL [6, 7]
Soman (GD)	$C_7H_{16}FO_2P$	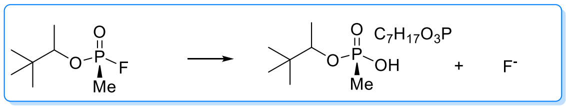		OPAA-immobilized MOF [10]; MOF (UiO-66-NH_2) [11–13]

VX	$C_{11}H_{26}NO_2PS$	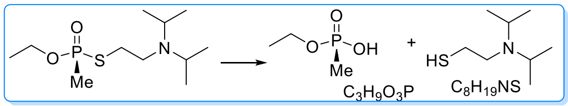	RSDL [6, 7]; MOF (UiO-66-NH_2) [11–13]
Paraoxon (POX) Ethyl	$C_{10}H_{14}NO_6P$	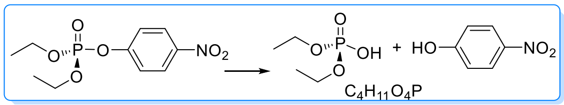	Oxime/HA conjugated PAMAM dendrimer [14, 15]; MOF (UiO-66-NH_2) [11–13]; PTE-encapsulated liposome [16]; La (catecholate) polymer [17]; CeO_2 [18]
Parathion Methyl	$C_8H_{10}NO_5PS$	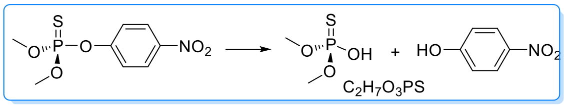	Cu (II)-bipyridyl polymer [19]; TiO_2 [20]
Fenitrothion	$C_9H_{12}NO_5PS$	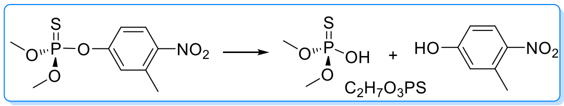	Oxime, HA liposome [21–24]
Chlorpyrifos	$C_9H_{11}C_{13}NO_3PS$	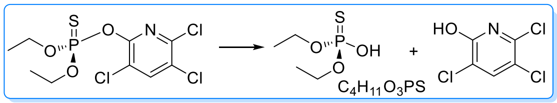	Ag-ZnO [25]; CdS QD [26]
Dichlorvos	$C_4H_7Cl_2O_4P$	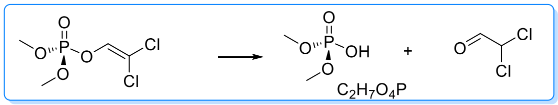	MSN [27]

HA = hydroxamic acid; MSN = mesoporous silica nanoparticle; MOF = metal organic framework; OPAA = organophosphorus acid anhydrolase; PTE = phosphotriesterase; PAMAM = poly(amidoamine); QD = quantum dot; RSDL = reactive skin decontamination lotion; SLN = solid lipid nanoparticle

References

1. Choi, S. K.; Thomas, T. P.; Leroueil, P. R.; Kotlyar, A.; Van Der Spek, A. F. L.; Baker, J. R. Specific and Cooperative Interactions between Oximes and PAMAM Dendrimers as Demonstrated by ^{1}H NMR Study. *J. Phys. Chem. B* **2012**, *116*, 10387–10397.
2. Choi, S. K.; Leroueil, P.; Li, M.-H.; Desai, A.; Zong, H.; Van Der Spek, A. F. L.; Baker Jr, J. R. Specificity and Negative Cooperativity in Dendrimer–Oxime Drug Complexation. *Macromolecules* **2011**, *44*, 4026–4029.
3. Pashirova, T. N.; Braïki, A.; Zueva, I. V.; Petrov, K. A.; Babaev, V. M.; Burilova, E. A.; Samarkina, D. A.; Rizvanov, I. K.; Souto, E. B.; Jean, L.; Renard, P.-Y.; Masson, P.; Zakharova, L. Y.; Sinyashin, O. G. Combination delivery of two oxime-loaded lipid nanoparticles: Time-dependent additive action for prolonged rat brain protection. *J. Controlled Release* **2018**, *290*, 102–111.
4. Pashirova, T. N.; Zueva, I. V.; Petrov, K. A.; Babaev, V. M.; Lukashenko, S. S.; Rizvanov, I. K.; Souto, E. B.; Nikolsky, E. E.; Zakharova, L. Y.; Masson, P.; Sinyashin, O. G. Nanoparticle-Delivered 2-PAM for Rat Brain Protection against Paraoxon Central Toxicity. *ACS Appl. Mater. Interfaces* **2017**, *9*, 16922–16932.
5. Mukherjee, J.; Wong, P. T.; Tang, S.; Gam, K.; Coulter, A.; Baker, J. R.; Choi, S. K. Mechanism of Cooperativity and Nonlinear Release Kinetics in Multivalent Dendrimer-Atropine Complexes. *Mol. Pharmaceutics* **2015**, *12*, 4498–4508.

6. Bjarnason, S.; Mikler, J.; Hill, I.; Tenn, C.; Garrett, M.; Caddy, N.; Sawyer, T. Comparison of Selected Skin Decontaminant Products and Regimens Against VX In Domestic Swine. *Hum. Exp. Toxicol.* **2008**, *27*, 253–261.
7. Fentabil, M.; Gebremedhin, M.; Purdon, J. G.; Cochrane, L.; Goldman, V. S. Degradation of Pesticides with RSDL® (Reactive Skin Decontamination Lotion Kit) Lotion: LC–MS Investigation. *Toxicol. Lett.* **2018**, *293*, 241–248.
8. Tang, S.; Wong, P. T.; Cannon, J.; Yang, K.; Bowden, S.; Bhattacharjee, S.; O'Konek, J. J.; Choi, S. K. Hydrophilic Scaffolds of Oxime as the Potent Catalytic Inactivator of Reactive Organophosphate. *Chem.-Biol. Interact.* **2019**, *297*, 67–79.
9. Wong, P.; Bhattacharjee, S.; Cannon, J.; Tang, S.; Yang, K.; Bowden, S.; Varnau, V.; O'Konek, J. J.; Choi, S. K. Reactivity and Mechanism of α-Nucleophile Scaffolds as Catalytic Organophosphate Scavengers. *Org. Biomol. Chem.* **2019**, *17*, 3951–3963.
10. Li, P.; Moon, S.-Y.; Guelta, M. A.; Lin, L.; Gómez-Gualdrón, D. A.; Snurr, R. Q.; Harvey, S. P.; Hupp, J. T.; Farha, O. K. Nanosizing a Metal–Organic Framework Enzyme Carrier for Accelerating Nerve Agent Hydrolysis. *ACS Nano* **2016**, *10*, 9174–9182.
11. de Koning, M. C.; van Grol, M.; Breijaert, T. Degradation of Paraoxon and the Chemical Warfare Agents VX, Tabun, and Soman by the Metal–Organic Frameworks UiO-66-NH2, MOF-808, NU-1000, and PCN-777. *Inorg. Chem.* **2017**, *56*, 11804–11809.
12. Katz, M. J.; Moon, S.-Y.; Mondloch, J. E.; Beyzavi, M. H.; Stephenson, C. J.; Hupp, J. T.; Farha, O. K. Exploiting parameter space in MOFs: a 20-fold enhancement of phosphate-ester hydrolysis with UiO-66-NH2. *Chem. Sci.* **2015**, *6*, 2286–2291.
13. Katz, M. J.; Mondloch, J. E.; Totten, R. K.; Park, J. K.; Nguyen, S. T.; Farha, O. K.; Hupp, J. T. Simple and Compelling Biomimetic Metal–Organic Framework Catalyst for the Degradation of Nerve Agent Simulants. *Angew. Chem., Int. Ed.* **2014**, *53*, 497–501.
14. Bharathi, S.; Wong, P. T.; Desai, A.; Lykhytska, O.; Choe, V.; Kim, H.; Thomas, T. P.; Baker, J. R.; Choi, S. K. Design and Mechanistic Investigation of Oxime-conjugated PAMAM Dendrimers As the Catalytic Scavenger of Reactive Organophosphate. *J. Mater. Chem. B* **2014**, *2*, 1068–1078.
15. Wong, P. T.; Tang, S.; Cannon, J.; Yang, K.; Harrison, R.; Ruge, M.; O'Konek, J. J.; Choi, S. K. Shielded α-Nucleophile Nanoreactor for Topical Decontamination of Reactive Organophosphate. *ACS Appl. Mater. Interfaces* **2020**, *12*, 33500–33515.
16. Alves, N. J.; Moore, M.; Johnson, B. J.; Dean, S. N.; Turner, K. B.; Medintz, I. L.; Walper, S. A. Environmental Decontamination of a Chemical Warfare Simulant Utilizing a Membrane Vesicle-Encapsulated Phosphotriesterase. *ACS Appl. Mater. Interfaces* **2018**, *10*, 15712–15719.
17. Totten, R. K.; Weston, M. H.; Park, J. K.; Farha, O. K.; Hupp, J. T.; Nguyen, S. T. Catalytic Solvolytic and Hydrolytic Degradation of Toxic Methyl Paraoxon with La(catecholate)-Functionalized Porous Organic Polymers. *ACS Catal.* **2013**, *3*, 1454–1459.
18. Salerno, A.; Devers, T.; Bolzinger, M.-A.; Pelletier, J.; Josse, D.; Briançon, S. In Vitro Skin Decontamination of the Organophosphorus Pesticide Paraoxon with Nanometric Cerium Oxide CeO2. *Chem.-Biol. Interact.* **2017**, *267*, 57–66.
19. Hartshorn, C. M.; Singh, A.; Chang, E. L. Metal-chelator polymers as organophosphate hydrolysis catalysts. *J. Mater. Chem.* **2002**, *12*, 602–605.
20. Konstantinou, I. K.; Sakellarides, T. M.; Sakkas, V. A.; Albanis, T. A. Photocatalytic Degradation of Selected s-Triazine Herbicides and Organophosphorus Insecticides over Aqueous TiO2 Suspensions. *Environ. Sci. Technol.* **2001**, *35*, 398–405.
21. Han, X.; Balakrishnan, V. K.; vanLoon, G. W.; Buncel, E. Degradation of the Pesticide Fenitrothion as Mediated by Cationic Surfactants and α-Nucleophilic Reagents. *Langmuir* **2006**, *22*, 9009–9017.
22. Singh, N.; Karpichev, Y.; Sharma, R.; Gupta, B.; Sahu, A. K.; Satnami, M. L.; Ghosh, K. K. From α-nucleophiles to functionalized aggregates: exploring the reactivity of hydroxamate ion towards esterolytic reactions in micelles. *Org. Biomol. Chem.* **2015**, *13*, 2827–2848.
23. Kandpal, N.; Dewangan, H. K.; Nagwanshi, R.; Ghosh, K. K.; Satnami, M. L. Micellar-accelerated hydrolysis of organophosphate and thiophosphates by pyridine oximate. *Int. J. Chem. Kinet.* **2018**, *50*, 827–835.
24. Gonçalves, L. M.; Kobayakawa, T. G.; Zanette, D.; Chaimovich, H.; Cuccovia, I. M. Effects of Micelles and Vesicles on the Oximolysis of p-Nitrophenyl Diphenyl Phosphate: A Model System for Surfactant-Based Skin-Defensive Formulations against Organophosphates. *J. Pharm. Sci. (Philadelphia, PA, U. S.)* **2009**, *98*, 1040–1052.
25. Choudhary, M. K.; Kataria, J.; Bhardwaj, V. K.; Sharma, S. Green biomimetic preparation of efficient Ag–ZnO heterojunctions with excellent photocatalytic performance under solar light irradiation: a novel biogenic-deposition-precipitation approach. *Nanoscale Adv.* **2019**, *1*, 1035–1044.

26. Dey, P. C.; Das, R. Ligand free surface of CdS nanoparticles enhances the energy transfer efficiency on interacting with Eosin Y dye – Helping in the sensing of very low level of chlorpyrifos in water. *Spectrochim. Acta, Part A* **2019**, *207*, 156–163.
27. Xu, P.; Guo, S.; Yu, H.; Li, X. Mesoporous Silica Nanoparticles (MSNs) for Detoxification of Hazardous Organophorous Chemicals. *Small* **2014**, *10*, 2404–2412.
